# Chemically reactive MHD micropolar nanofluid flow with velocity slips and variable heat source/sink

**DOI:** 10.1038/s41598-020-77615-9

**Published:** 2020-12-01

**Authors:** Abdullah Dawar, Zahir Shah, Poom Kumam, Hussam Alrabaiah, Waris Khan, Saeed Islam, Nusrat Shaheen

**Affiliations:** 1 Department of Mathematics, Abdul Wali Khan University, Mardan, 23200 Khyber Pakhtunkhwa Pakistan; 2 Department of Mathematics, University of Lakki Marwat, Lakki Marwat, 28420 Khyber Pakhtunkhwa Pakistan; 3 Center of Excellence in Theoretical and Computational Science (TaCS-CoE), SCL 802 Fixed Point Laboratory, Science Laboratory Building, King Mongkut’s University of Technology Thonburi (KMUTT), 126 Pracha-Uthit Road, Bang Mod, Thung Khru, Bangkok, 10140 Thailand; 4 KMUTTFixed Point Research Laboratory, Room SCL 802 Fixed Point Laboratory, Science Laboratory Building, Department of Mathematics, Faculty of Science, King Mongkut’s University of Technology Thonburi (KMUTT), 126 Pracha-Uthit Road, Bang Mod, Thung Khru, Bangkok, 10140 Thailand; 5 Department of Medical Research, China Medical University Hospital, China Medical University, Taichung, 40402 Taiwan; 6 College of Engineering, Al Ain University, 64141 Al Ain, United Arab Emirates; 7 Department of Mathematics, Tafila Technical University, Tafila, 66110 Jordan; 8 Department of Mathematics and Statistics, Hazara University, Mansehra, 21120 Khyber Pakhtunkhwa Pakistan; 9 Faculty of Mathematics and Statistics, Ton Duc Thang University, Ho Chi Minh City, 70000 Vietnam; 10 Informetrics Research Group, Ton Duc Thang University, Ho Chi Minh City, 70000 Vietnam; 11 Institute of Chemistry, Gulab Devi Educational Complex, Lahore, 54000 Punjab Pakistan

**Keywords:** Fluid dynamics, Mechanical engineering, Applied mathematics, Nanoscale materials

## Abstract

The two-dimensional electrically conducting magnetohydrodynamic flow of micropolar nanofluid over an extending surface with chemical reaction and secondary slips conditions is deliberated in this article. The flow of nanofluid is treated with heat source/sink and nonlinear thermal radiation impacts. The system of equations is solved analytically and numerically. Both analytical and numerical approaches are compared with the help of figures and tables. In order to improve the validity of the solutions and the method convergence, a descriptive demonstration of residual errors for various factors is presented. Also the convergence of an analytical approach is shown. The impacts of relevance parameters on velocity, micro-rotation, thermal, and concentration fields for first- and second-order velocity slips are accessible through figures. The velocity field heightens with the rise in micropolar, micro-rotation, and primary order velocity parameters, while other parameters have reducing impact on the velocity field. The micro-rotation field reduces with micro-rotation, secondary order velocity slip, and micropolar parameters but escalates with the primary order velocity slip parameter. The thermal field heightens with escalating non-uniform heat sink/source, Biot number, temperature ratio factor, and thermal radiation factor. The concentration field escalates with the increasing Biot number, while reduces with heightening chemical reaction and Schmidt number. The assessment of skin factor, thermal transfer, and mass transfer are calculated through tables.

## Introduction

The uses of nanotechnology in the field of engineering and technologies are electric circuits, solar cells, food processing, chemical sensors, batteries, fuels, ethanol, etc. Such applications and advantages have endorsed the researchers’ interest in the field of nanotechnology. Nowadays, the investigators are analyzing a new subclass of nanotechnology called nanofluids. Initially, the nanofluid was determined by Choi^1^. Rashidi et al.^2^ explored the heat transfer analysis of nanofluid. Bahiraei and Hangi^3^ investigated the transfer of heat by nanofluids with a magnetic influence. Ghasemian et al.^4^ tested the nanofluid heat transfer with alternating and constant magnetic field. Ellahi et al.^5^ intentioned the nanofluid stream of the boundary layer. Xuan et al.^6^ assessed heat propagation in the nanofluid flow. Sheikholeslami et al.^7^ probed the Coulomb force influence on the heat transfer of a nanofluid. Alsabery et al.^8^ presented the nanofluid flow in an inclined enclosure with a porous medium. Sheikholeslami^9^ probed the flow of nanofluid in porous media. Sheikholeslami^10^ analyzed the nanofluid flow with a magnetic field effect. Hassan et al.^11^ observed the flow of nanofluid in a porous medium with a convective heat exchange. Dawar et al.^12^ studied the flow of nanofluid with thermal energy and energy source. Khan et al.^13^ tested the convective boundary layer nanofluid flow through mass and heat distribution. Sheikholeslami et al.^14^ monitored the thermal conduction to a magnetic nanofluid. Shah et al.^15^ tested the nanofluid thin film flow with a nonlinear thermal radiation. Shah et al.^16^ looked at the coupled stress nanofluid flow with the Cattaneo heat design. Sohail et al.^17^ presented the ferrofluid videography valuation in drug targeting. Dawar et al.^18^ inspected the nanofluid flow with thermophoretic and Brownian motion influences. Majeed et al.^19^ probed the nanofluid heat transmission with heat source and thermal energy effects. Dawar et al.^20^ investigated MHD nanofluid with dissipation impact. Sheikholeslami et al.^21^ numerically checked the flow of ferrofluid using porous media. Sajjad et al.^22^ offered the flow of nanofluid with thermophoretic and Brownian motion influences using Darcy-Forchheimer relation. Dawar et al.^23^ probed the thin film nanofluid flow with thermal energy using the Darcy-Forchheimer relation. Alamri et al.^24^ monitored the flow of nanofluid in a porous medium with slip conditions. Ahmad et al.^25^ offered the couple stress nanofluid flow with the Cattaneo heat model using the Darcy-Forchheimer relation.

The situation of stretching performs a significance role in boundary layer flow examination due to its remarkable results in the area of engineering and industries for instance, paper production, polymer engineering, metallic beds cooling, wire drawing, hot rolling, plastic sheets extraction, glass formation, etc. Preceding the stretching phenomenon, Crane^26^ was the discoverer who introduced the fluid flow over the extending plate. Hayat et al.^27^ examined the micropolar fluid flow. Najib et al.^28^ investigated the stagnation point flow with chemical reaction. Babu et al.^29^ scrutinized the micropolar fluid flow’s stagnation point with suction impact. Soid et al.^30^ presented the heat transmission of a fluid flow. Makinde et al.^31^ probed the boundary layer nanofluid flow with a magnetic field. Mabood et al.^32^ scrutinized the nanofluid flow with melting absorption/generation impacts.

Rarefaction influences should always be assumed in order to accurately design a microsystem. Rarefaction impacts become huge as the distinctive length of a system moves toward the molecular mean free pathway of the liquid inside the framework. The Knudsen number, $K_{n}$, characterized as the proportion of the molecular mean free way to the trademark length of the framework, is regularly used to exactly measure the impacts of rarefaction^33^. For continuum flow,$K_{n} \le 0.01$ rarefaction impacts are immaterial and traditional preservation conditions are utilized. $0.1 \le K_{n} \le 10.0$, extremely infrequent flows are part of the transitional system. $K_{n} \ge 10.0$, the open molecular system. The presumption that because a liquid rigidly adhere to a solid boundary and this called no-slip boundary condition was shown to be ineffective in a variety of cases, like: the high molecular liquid flows, micro-channel flows or dynamics of thin fluids. Slip conditions are very important for the nanofluids studies. Eggs yolk, oil, and liquid combination, grease, polymer solutions, etc. are the application containing slip conditions influences. A substantial majority of models were discussed to characterize the slip that actually happens at a solid boundary. In 1827, Navier^34^ presented the fluid motion through a sheet with a slip condition. Fang et al.^35^ investigated the flow of viscous fluid in a second-order slip state. Beg et al.^36^ tested the magnetic flow in slippery conditions. Martin and Boyd^37^ tested the convection in a boundary layer flow with slip condition. Ibrahim et al.^38^ proposed a thermal transfer of a magnetohydrodynamic micropolar fluid with a second-order slip state. Maboob et al.^39^ explored the heat transmission of stagnation point flow with second-order slip conditions. Other relevant analyses are mentioned in^40–43^. Lund et al.^44^ presented the MHD micropolar fluid flow with joule heating and viscous dissipation influences over exponentially shrinking sheet. Yasmin et al.^45^ investigated the thermal and mass transmission in MHD micropolar fluid flow over a stretching surface. Kumar et al.^46^ investigated the MHD micropolar fluid flow over a stretching sheet with heat flux model. The MHD stagnation point flow of micropolar fluid over a convective surface with nonlinear radiation influence was analyzed by Kumar et al.^47^. Kumar et al.^43^ analyzed the MHD first and second orders slips flow of micropolar fluid over a convective surface. Kumar et al.^48,49^ investigated the micropolar fluid flow with heat flux model under the influence of magnetic field, thermophoresis and Brownian motion using coagulated and stretching surfaces. Further studies of Kumar et al. can be seen in^50–53^.

The key explanation for the considerable attention paid to the analysis of micropolar fluid flows is the uses and applications in manufacturing processes, including: animal blood, liquid crystal solidification, bath metal plate cooling, suspension and colloidal solutions, polymer fluid extrusion, and exotic lubricants. In order to present the current work in the field of micropolar fluids, we present the flow of micropolar nanofluid over an extending sheet in the presence of first and second orders velocity slip conditions with chemical reaction. The system of equations is solved analytically and numerically. The effects of developed factors on the nanofluid flow are presented through graphs and deliberated their features.

## Problem formulation

**Figure 1:**
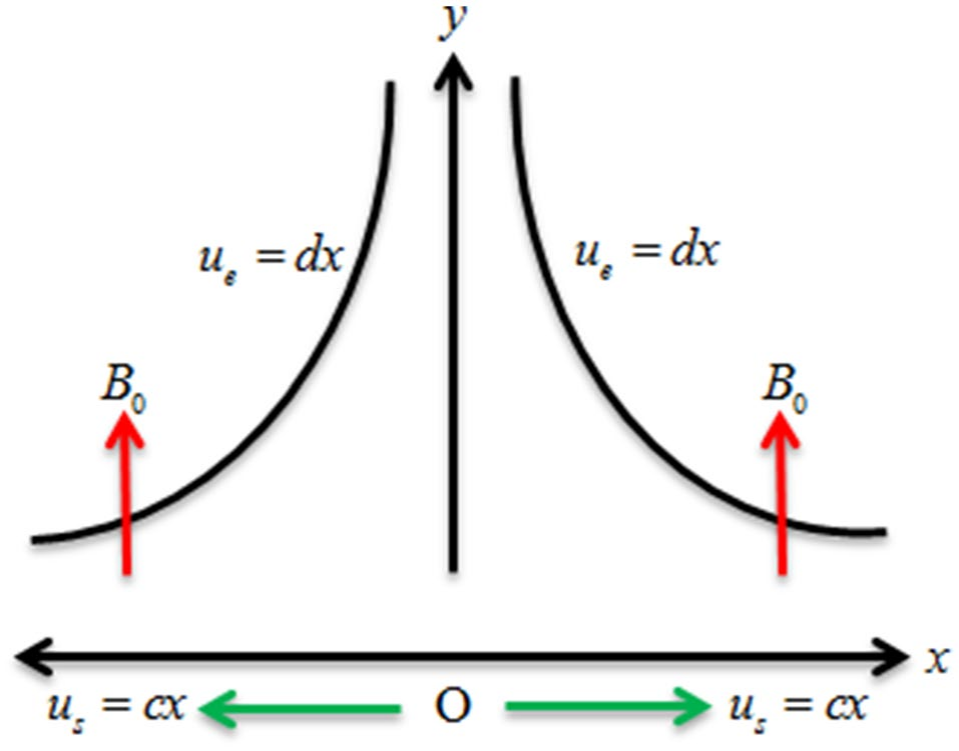
Geometry of the problem.

We considered the electrically accompanying magnetohydrodynamic flow of micropolar fluid over an extending sheet with chemical reaction and secondary slips conditions. The nanofluid flow is treated with heat source/sink and nonlinear thermal radiation. The nanofluid flow is considered in 2D coordinates system. The *x*-axis is considered along the nanofluid flow and *y*-axis is considered vertical to the nanofluid flow. The strength of the magnetic field is taken vertically to the nanofluid flow. The velocities are $u_{s} = cx$ and $u_{e} = dx$ where $c > 0$ and $d > 0$ are constants as expressed in Fig. 1.

The proposed model leads to the following equations^54–56^:

1 $$\frac{\partial v}{{\partial y}} + \frac{\partial u}{{\partial x}} = 0,$$

2 $$\rho \left( {v\frac{\partial u}{{\partial y}} + u\frac{\partial u}{{\partial x}}} \right) = \kappa \left( {\frac{{\partial^{2} u}}{{\partial y^{2} }} + \frac{\partial N}{{\partial y}}} \right) + \mu \frac{{\partial^{2} u}}{{\partial y^{2} }} - \sigma B_{0}^{2} u,$$

3 $$\rho j\left( {v\frac{\partial N}{{\partial y}} + u\frac{\partial N}{{\partial x}}} \right) = - \kappa \left( {2N + \frac{\partial u}{{\partial y}}} \right) + \Gamma \frac{{\partial^{2} N}}{{\partial y^{2} }},$$

4 $$\rho c_{p} \left( {v\frac{\partial T}{{\partial y}} + u\frac{\partial T}{{\partial x}}} \right) = k\frac{{\partial^{2} T}}{{\partial y^{2} }} - \frac{{\partial q_{f} }}{\partial y} + q^{\prime\prime\prime},$$

5 $$u\frac{\partial C}{{\partial x}} + v\frac{\partial C}{{\partial y}} = D\frac{{\partial^{2} C}}{{\partial y^{2} }} - K_{1} \left( {C - C_{\infty } } \right),$$

where $\Gamma$, $q_{f}$ and $q^{\prime\prime\prime}$ are defined as

6 $$\begin{gathered} \Gamma = \left( {\mu + \frac{\kappa }{2}} \right)j = \left( {1 + \frac{\alpha }{2}} \right)\mu j,\,\,q_{f} = - \frac{{4\sigma^{*} }}{{3k^{*} }}\frac{{\partial T^{4} }}{\partial y} = - \frac{{16\sigma^{*} }}{{3k^{*} }}T^{3} \frac{\partial T}{{\partial y}}, \hfill \\ q^{\prime\prime\prime} = \frac{{k\left( {T_{s} - T_{\infty } } \right)u_{s} }}{x\upsilon }\left( {A^{*} f^{\prime} + B^{*} \frac{{\left( {T - T_{\infty } } \right)}}{{\left( {T_{s} - T_{\infty } } \right)}}} \right), \hfill \\ \end{gathered}$$

Equation (4) is reduced as:

7 $$\rho c_{p} \left( {v\frac{\partial T}{{\partial y}} + u\frac{\partial T}{{\partial x}}} \right) = \frac{{16\sigma^{*} }}{{3k^{*} }}\frac{\partial }{\partial y}\left( {T^{3} \frac{\partial T}{{\partial y}}} \right) + k\frac{{\partial^{2} T}}{{\partial y^{2} }} + \frac{{k\left( {T_{s} - T_{\infty } } \right)u_{s} }}{x\upsilon }\left( {A^{*} f^{\prime} + B^{*} \frac{{\left( {T - T_{\infty } } \right)}}{{\left( {T_{s} - T_{\infty } } \right)}}} \right),$$

with boundary conditions^56^:

8 $$\begin{gathered} u = u_{s} + u_{{{\text{slip}}}} ,\,\,v = 0,\,\,N = - Mr\frac{\partial u}{{\partial y}},\,\, - \frac{\partial T}{{\partial y}} = \frac{{h_{f} }}{k}\left( {T_{s} - T} \right),\,\, - \frac{\partial C}{{\partial y}} = \frac{{h_{s} }}{D}\left( {C_{s} - C} \right)\,\,{\text{at }}y = 0, \hfill \\ u \to u_{e} ,\,\,N \to 0,\,\,T \to T_{\infty } ,\,\,C \to C_{\infty } \,\,{\text{as }}y \to \infty . \hfill \\ \end{gathered}$$

The secondary velocity slip model is considered as^56^:

9 $$u_{{{\text{slip}}}} = \frac{2}{3}\lambda \left( {\frac{{3 - al^{2} }}{a} - \frac{3}{2}\left( {\frac{{1 - l^{2} }}{{K_{n} }}} \right)} \right)\frac{\partial u}{{\partial y}} - \frac{1}{4}\lambda^{2} \left( {l^{4} + \frac{{2\left( {1 - l^{2} } \right)}}{{K_{n}^{2} }}} \right)\frac{{\partial^{2} u}}{{\partial y^{2} }} = P\frac{\partial u}{{\partial y}} + Q\frac{{\partial^{2} u}}{{\partial y^{2} }},$$

Following the overhead equations, $u,\,\,v$ are the velocity constituents along $x$- and $y$-coordinates, respectively, $\kappa$ represents the vertex viscosity, $B_{0}$ is the magnetic field strength,$\mu$ indicates the dynamic viscosity, $\rho$ designates the density, $\sigma$ represents the electrical conductivity, $\rho c_{p}$ denotes the heat capacitance, $k$ indicates the thermal conductivity, $D$ specifies the diffusion coefficient, $K_{1}$ represents the reaction rate, $N$ represents the micro-rotation velocity, $Mr$ represents the micro-rotation parameter, $j = {\upsilon \mathord{\left/ {\vphantom {\upsilon c}} \right. \kern-\nulldelimiterspace} c}$ represents the micro-inertia density, $\alpha = {\kappa \mathord{\left/ {\vphantom {\kappa \mu }} \right. \kern-\nulldelimiterspace} \mu }$ indicates the material parameter, $T$, $T_{s}$ and $T_{\infty }$ represent the temperature, temperature at the surface and temperature away from the surface, correspondingly, $C$, $C_{s}$ and $C_{\infty }$ represent the concentration, concentration at the surface and concentration away from the surface, correspondingly, $h_{f}$ and $h_{s}$ indicate the convective heat and mass transfer coefficients respectively, $A^{*}$ and $B^{*}$ are the non-uniform heat source and sink parameters respectively, $P$ and $Q$ are constants, $K_{n}$ indicates the Knudsen number, $a\left( {0 \le a \le 1} \right)$ signifies the momentum coefficient, and $\lambda$ indicates the molecular free path, $l = {\text{min}}\left( {\frac{1}{{K_{n} }},\,1} \right)$ for all $K_{n}$.

The similarity transformations are defined as^54–56^:

10 $$\begin{gathered} \chi = \left( {c\upsilon } \right)^{{{\raise0.7ex\hbox{$1$} \!\mathord{\left/ {\vphantom {1 2}}\right.\kern-\nulldelimiterspace} \!\lower0.7ex\hbox{$2$}}}} xf,\,\,\,u = \frac{\partial \chi }{{\partial y}},\,\,v = - \frac{\partial \chi }{{\partial x}},\,\,N = cx\left( {\frac{c}{\upsilon }} \right)^{{{\raise0.7ex\hbox{$1$} \!\mathord{\left/ {\vphantom {1 2}}\right.\kern-\nulldelimiterspace} \!\lower0.7ex\hbox{$2$}}}} g,\,\, \hfill \\ T = T_{\infty } \left( {1 + \left( {\theta_{w} - 1} \right)\theta } \right),\,\,\theta_{w} = \frac{{T_{s} }}{{T_{\infty } }},\,\,\phi \left( \xi \right) = \frac{{C - C_{\infty } }}{{C_{s} - C_{\infty } }},\,\,\xi = \left( {\frac{c}{\upsilon }} \right)^{{{\raise0.7ex\hbox{$1$} \!\mathord{\left/ {\vphantom {1 2}}\right.\kern-\nulldelimiterspace} \!\lower0.7ex\hbox{$2$}}}} y,\, \hfill \\ \end{gathered}$$

Equations (2), (3), (5) and (7) with boundary conditions (8) are reduced as:

11 $$\left( {1 + \alpha } \right)f^{\prime\prime\prime} + ff^{\prime\prime} - f^{{\prime}{2}} + \alpha g^{\prime} - Mf^{\prime} = 0,$$

12 $$\left( {1 + \frac{\alpha }{2}} \right)g^{\prime\prime} + fg^{\prime} - gf^{\prime} - 2\alpha g - \alpha f^{\prime\prime} = 0,$$

13 $$\theta^{\prime\prime} + \Pr f\theta^{\prime} + A^{*} f^{\prime} + B^{*} \theta + Rd\left\{ \begin{gathered} \theta^{\prime\prime} + \theta^{3} \theta^{\prime\prime}\left( {\theta_{w} - 1} \right)^{3} + 3\theta^{2} \theta^{{\prime}{2}} \left( {\theta_{w} - 1} \right)^{3} \hfill \\ + 3\theta^{2} \theta^{\prime\prime}\left( {\theta_{w} - 1} \right)^{2} + 6\theta \theta^{{\prime}{2}} \left( {\theta_{w} - 1} \right)^{2} \hfill \\ + 3\theta \theta^{\prime\prime}\left( {\theta_{w} - 1} \right) + 3\theta^{{\prime}{2}} \left( {\theta_{w} - 1} \right) \hfill \\ \end{gathered} \right\} = 0,$$

14 $$\phi^{\prime\prime} + Scf\phi^{\prime} - ScC_{r} \phi = 0,$$

Subject to:

15 $$\begin{gathered} f = 0,\,\,f^{\prime} = 1 + \delta f^{\prime\prime\prime} + \gamma f^{\prime\prime},\,\,\,g = - f^{\prime\prime}\,M_{r} ,\,\,\theta^{\prime} = - Bi_{1} \left( {1 - \theta } \right),\,\,\phi^{\prime} = - Bi_{2} \left( {1 - \phi } \right)\,\,{\text{at}}\,\,\xi = 0, \hfill \\ f^{\prime} \to \lambda ,\,\,g \to 0,\,\,\theta \to 0,\,\,\phi \to 0\,\,{\text{as}}\,\,\xi \to \infty \hfill \\ \end{gathered}$$

Here $M$ indicates the magnetic factor, $\alpha$ indicates the micropolar factor, $\Pr$ represents the Prandtl number, $Rd$ signifies the non-linear thermal energy factor, $M_{r}$ indicates the microrotation parameter, $C_{r}$ is chemical reaction parameter, $Bi_{1}$ and $Bi_{2}$ represent the Biot numbers, $\gamma$ and $\delta$ designate the velocity slip factors, and $\lambda$ indicates the stretching factor which are defined as

16 $$\begin{gathered} M = {{\sigma B_{0}^{2} } \mathord{\left/ {\vphantom {{\sigma B_{0}^{2} } {\rho c}}} \right. \kern-\nulldelimiterspace} {\rho c}},\,\,\Pr = \frac{{\mu c_{p} }}{k},\,\,Rd = \frac{{16\sigma^{*} T_{\infty }^{3} }}{{3kk^{*} }},\,\,Sc = \frac{\upsilon }{D},\,\,Bi_{1} = \frac{{h_{f} }}{k}\left( {\frac{\upsilon }{c}} \right)^{{{\raise0.7ex\hbox{$1$} \!\mathord{\left/ {\vphantom {1 2}}\right.\kern-\nulldelimiterspace} \!\lower0.7ex\hbox{$2$}}}} , \hfill \\ C_{r} = \frac{{K_{1} }}{c},\,\,Bi_{2} = \frac{{h_{s} }}{D}\left( {\frac{\upsilon }{c}} \right)^{{{\raise0.7ex\hbox{$1$} \!\mathord{\left/ {\vphantom {1 2}}\right.\kern-\nulldelimiterspace} \!\lower0.7ex\hbox{$2$}}}} ,\,\,\gamma = P\left( {\frac{c}{\upsilon }} \right)^{{{\raise0.7ex\hbox{$1$} \!\mathord{\left/ {\vphantom {1 2}}\right.\kern-\nulldelimiterspace} \!\lower0.7ex\hbox{$2$}}}} \left( { > 0} \right),\,\,\delta = Q\frac{c}{\upsilon }\left( { < 0} \right),\,\,\lambda = \frac{d}{c}. \hfill \\ \end{gathered}$$

The dimensionless form of the skin friction, couple stress, and Nusselt and Sherwood numbers are defined as:

17 $$\begin{gathered} \sqrt {{\text{Re}}_{x} } C_{f} = 2\left( {\alpha \left( {1 - Mr} \right) + 1} \right)f^{\prime \prime } \left( 0 \right),\,\,\,C_{s} = \left( {\frac{\alpha }{2} + 1} \right)g^{\prime}\left( 0 \right),\,\, \hfill \\ \,\frac{1}{{\sqrt {{\text{Re}}_{x} } }}Nu = - \left( {1 + Rd\left( {\theta_{w} } \right)^{3} } \right)\theta^{\prime}\left( 0 \right),\,\,\frac{1}{{\sqrt {{\text{Re}}_{x} } }}Sh = - \phi^{\prime}\left( 0 \right), \hfill \\ \end{gathered}$$

In which ${\text{Re}}_{x} = \frac{{cx^{2} }}{\upsilon }$ is Reynolds number.

## Solution by HAM

In understanding of (11–14) with (15), HAM is used with the following procedure.

Preliminary assumptions:

18 $$f_{0} (\xi ) = \frac{1}{{\left( {1 + \gamma - \delta } \right)}}\left( {1 - e^{ - \xi } } \right),\,\,g_{0} (\xi ) = - \frac{{M_{r} }}{{\left( {1 + \gamma - \delta } \right)}}e^{ - \xi } ,\,\,\theta_{0} (\xi ) = \frac{{Bi_{1} }}{{\left( {1 + Bi_{1} } \right)}}e^{ - \xi } {,}\,\,\phi_{0} (\xi ) = \frac{{Bi_{2} }}{{\left( {1 + Bi_{2} } \right)}}e^{ - \xi } .$$

Linear operators:

19 $$L_{f} \left( f \right) = \frac{{d^{3} f}}{{d\xi^{3} }} - \frac{df}{{d\xi }},\,\,{\text{L}}_{g} \left( g \right) = \frac{{d^{2} g}}{{d\xi^{2} }} - g,\,\,{\text{L}}_{\theta } \left( \theta \right) = \frac{{d^{2} \theta }}{{d\xi^{2} }} - \theta ,\,\,L_{\phi } \left( \phi \right) = \frac{{d^{2} \phi }}{{d\xi^{2} }} - \phi ,$$

with

20 $$L_{f} \left( {a_{1} + a_{2} e^{ - \xi } + a_{3} e^{\xi } } \right) = 0,{\text{ L}}_{g} \left( {a_{4} e^{ - \xi } + a_{5} e^{\xi } } \right) = 0,{\text{L}}_{\theta } \left( {a_{6} e^{ - \xi } + a_{7} e^{\xi } } \right) = 0,{\text{ L}}_{\phi } \left( {a_{8} e^{ - \xi } + a_{9} e^{\xi } } \right) = 0.$$

where $a_{i} (i = 1 - 9)$ are constants in general solution.

## Convergence analysis by HAM

**Figure 2:**
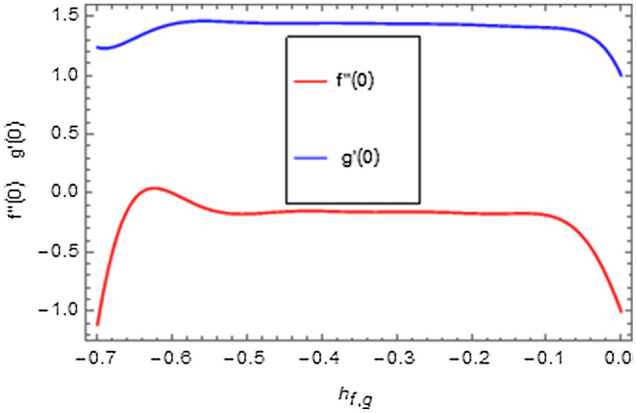
$\hbar$-curves for $f^{\prime}\left( \xi \right)$ and $g\left( \xi \right)$.

**Figure 3:**
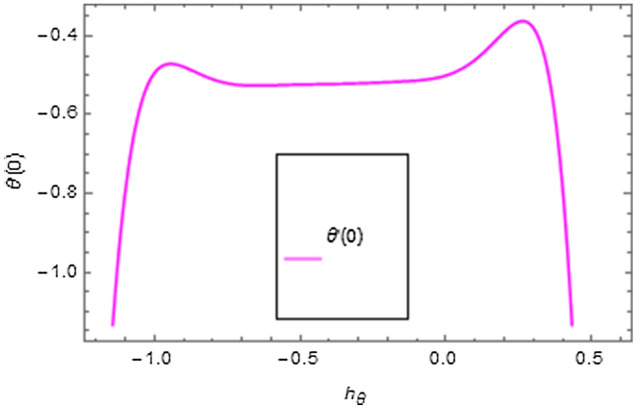
$$\hbar$$-curve for $$\theta \left( \xi \right)$$.

**Figure 4:**
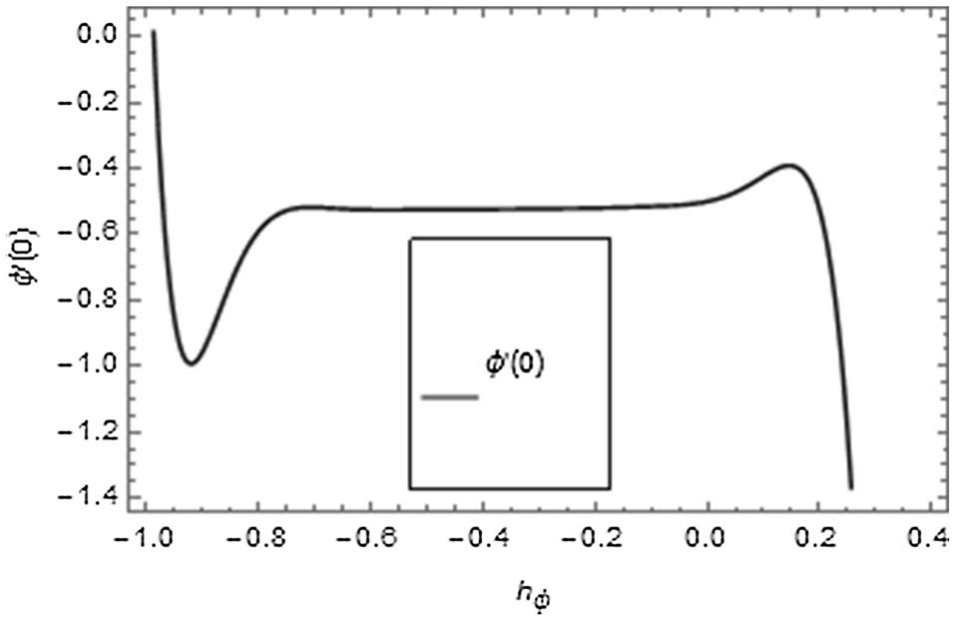
$$\hbar$$-curve for $$\phi \left( \xi \right)$$.

It is very well understood that the definition of homotopy ensures excellent versatility in interpreting the auxiliary factors ($\hbar_{f}$, $\hbar_{g}$, $\hbar_{\theta }$, $\hbar_{\phi }$) for regulating and modifying the series solutions convergence. In Figs. 2, 3, 4, $\hbar$-curves are displayed to interpret the appropriate values of $\hbar_{f}$, $\hbar_{g}$, $\hbar_{\theta }$ and $\hbar_{\phi }$. The acceptable ranges for the modeled problem are $- 0.54 \le \hbar_{f} \le - 0.08$, $- 0.58 \le \hbar_{g} \le - 0.04$, $- 0.8 \le \hbar_{\theta } \le 0.0$ and $- 0.75 \le \hbar_{\phi } \le 0.0$.

## Results and discussion

**Figure 5:**
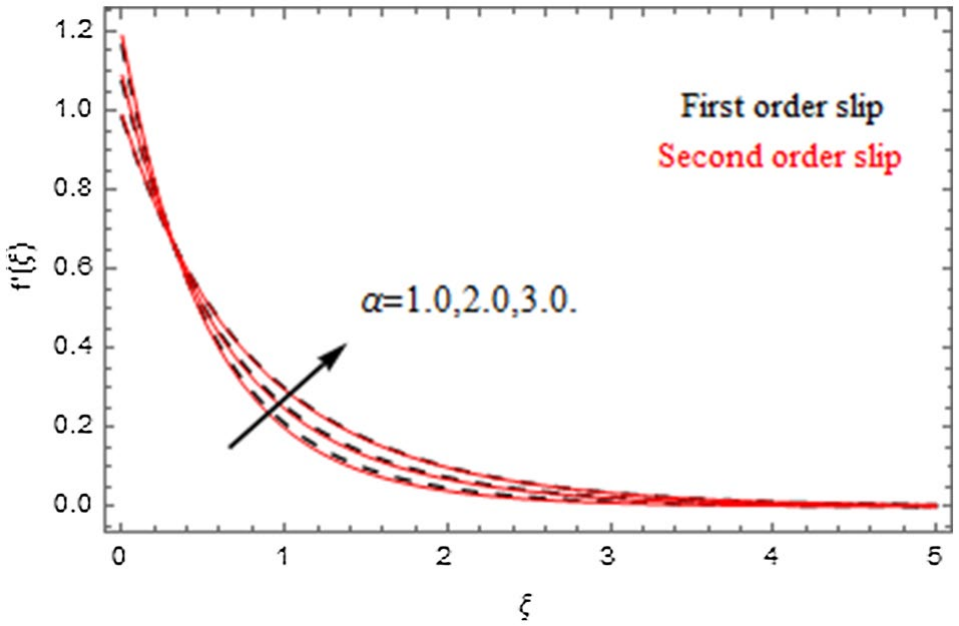
$$\alpha$$ versus $$f^{\prime}\left( \xi \right)$$.

**Figure 6:**
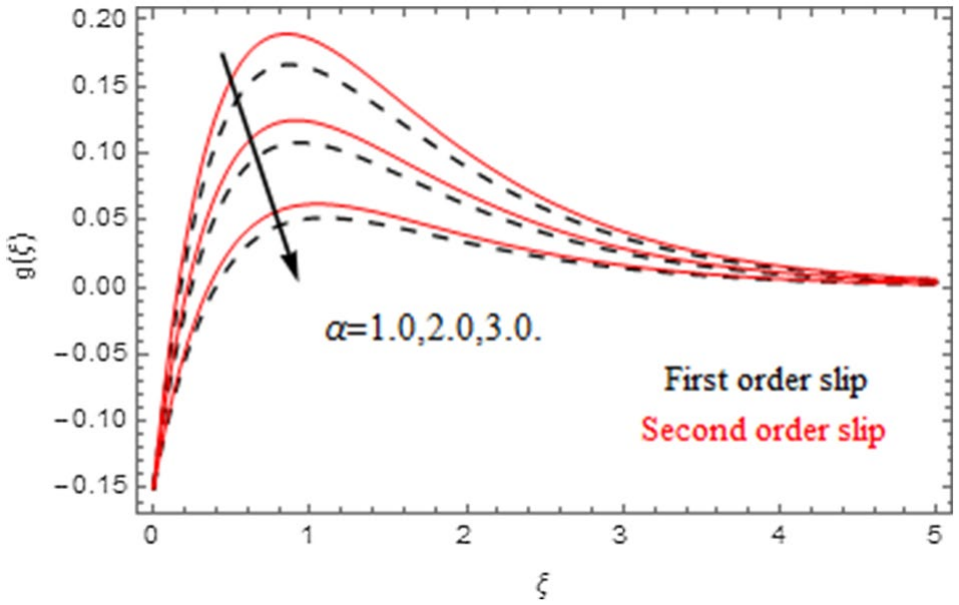
$$\alpha$$ versus $$g\left( \xi \right)$$.

**Figure 7:**
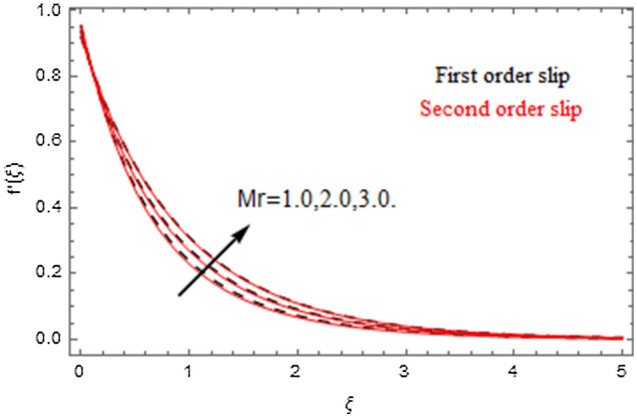
$$Mr$$ versus $$f^{\prime}\left( \xi \right)$$.

**Figure 8:**
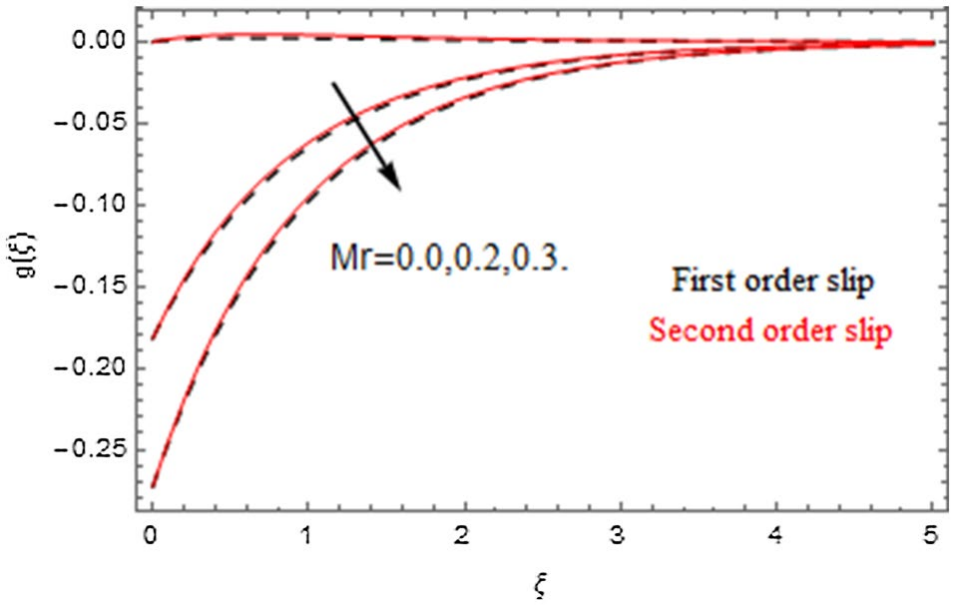
$$Mr$$ versus $$g\left( \xi \right)$$.

**Figure 9:**
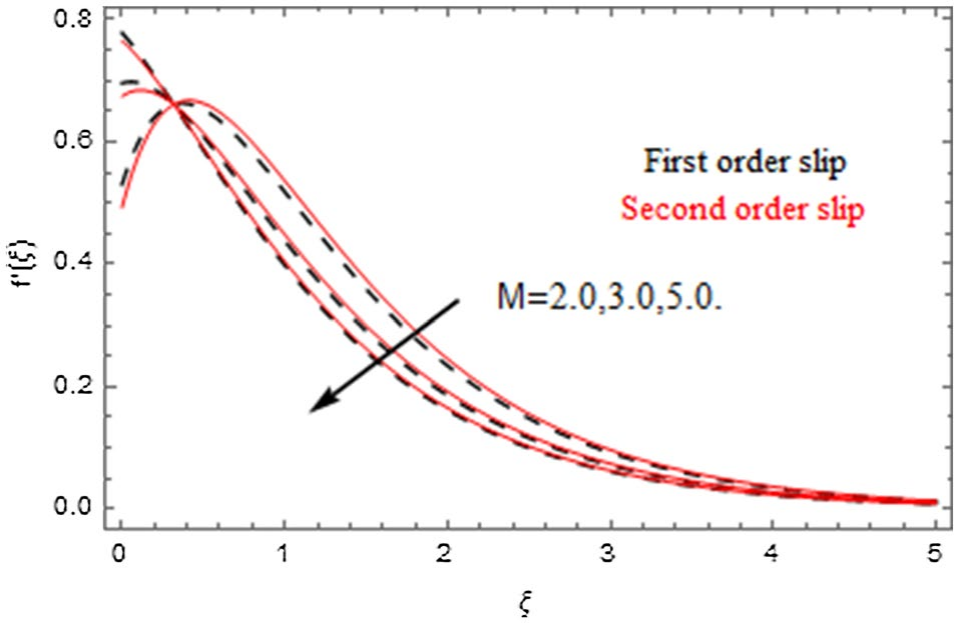
$$M$$ versus $$f^{\prime}\left( \xi \right)$$.

**Figure 10:**
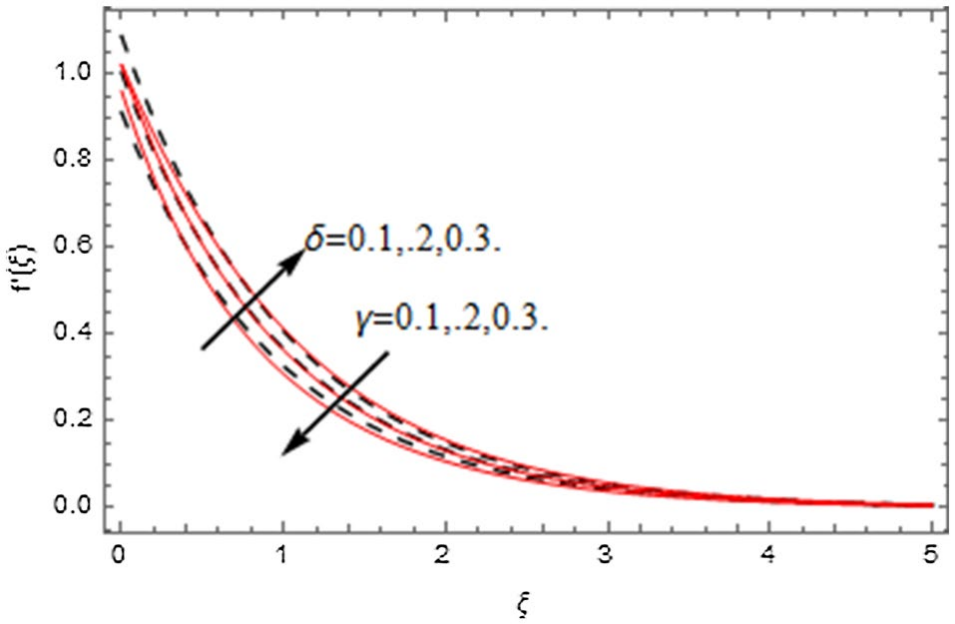
$$\delta$$ and $$\gamma$$ versus $$f^{\prime}\left( \xi \right)$$.

**Figure 11:**
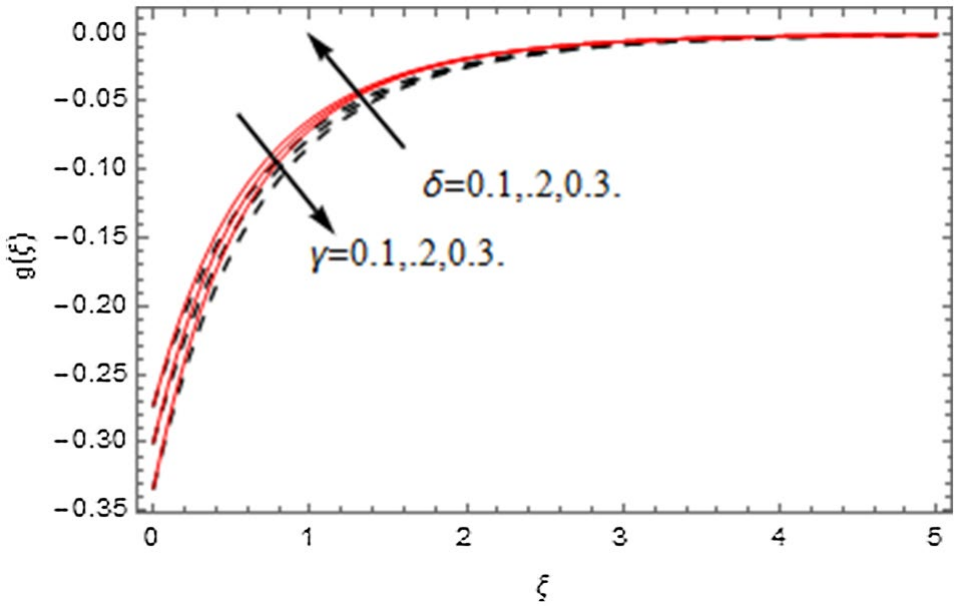
$$\delta$$ and $$\gamma$$ versus $$g\left( \xi \right)$$.

**Figure 12:**
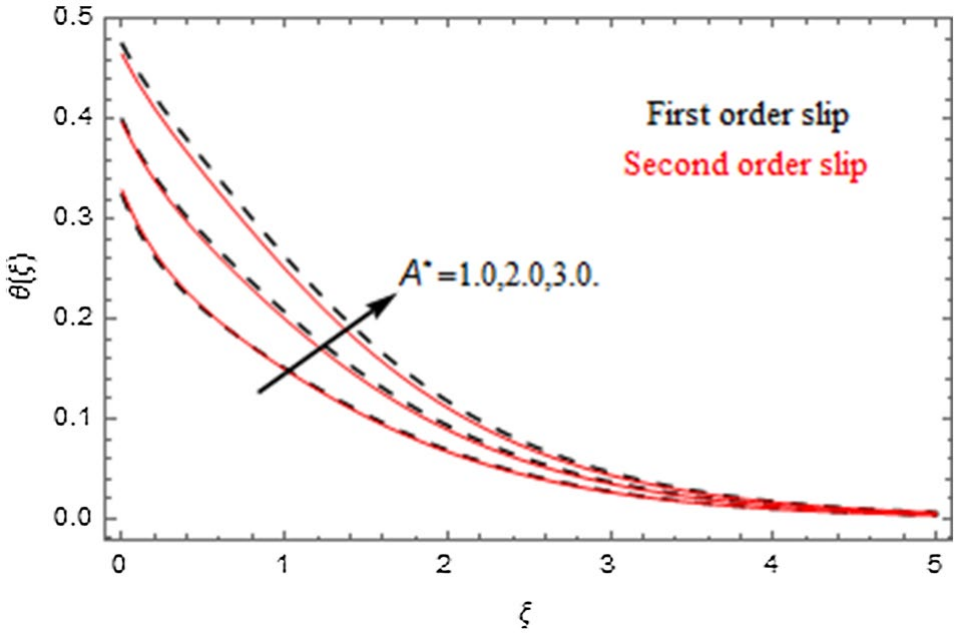
$$A^{*}$$ versus $$\theta \left( \xi \right)$$.

**Figure 13:**
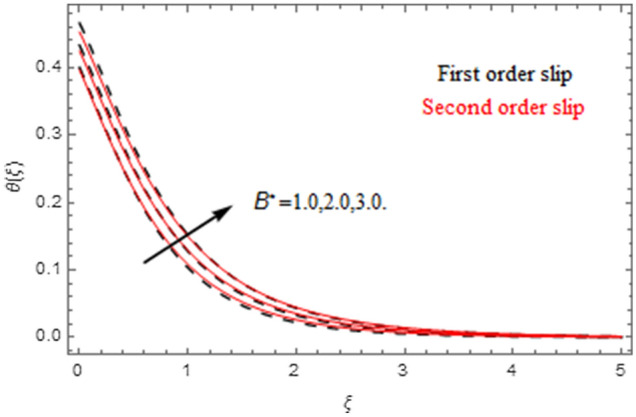
$$B^{*}$$ versus $$\theta \left( \xi \right)$$.

**Figure 14:**
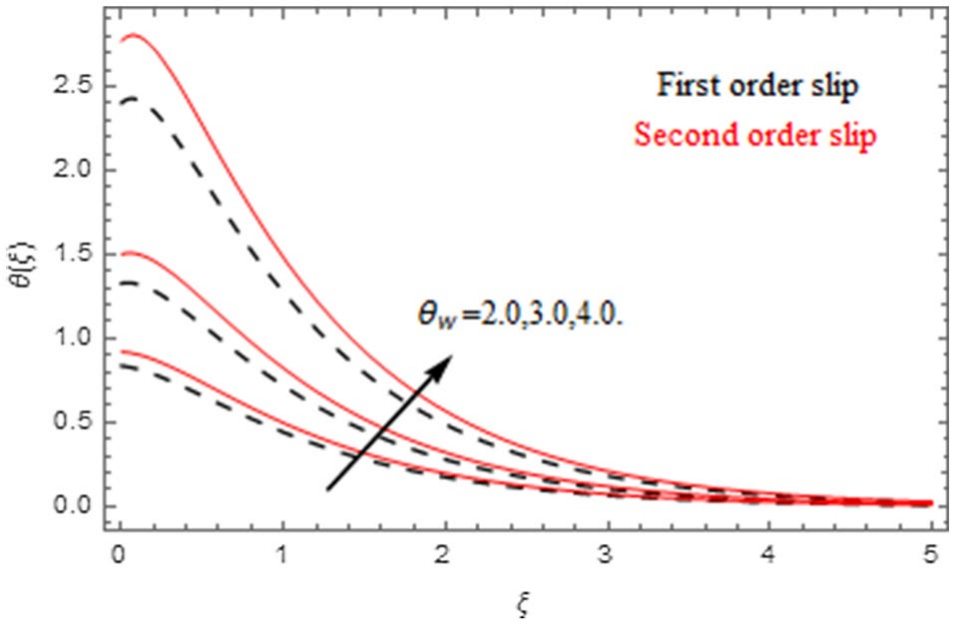
$$\theta_{w}$$ versus $$\theta \left( \xi \right)$$.

**Figure 15:**
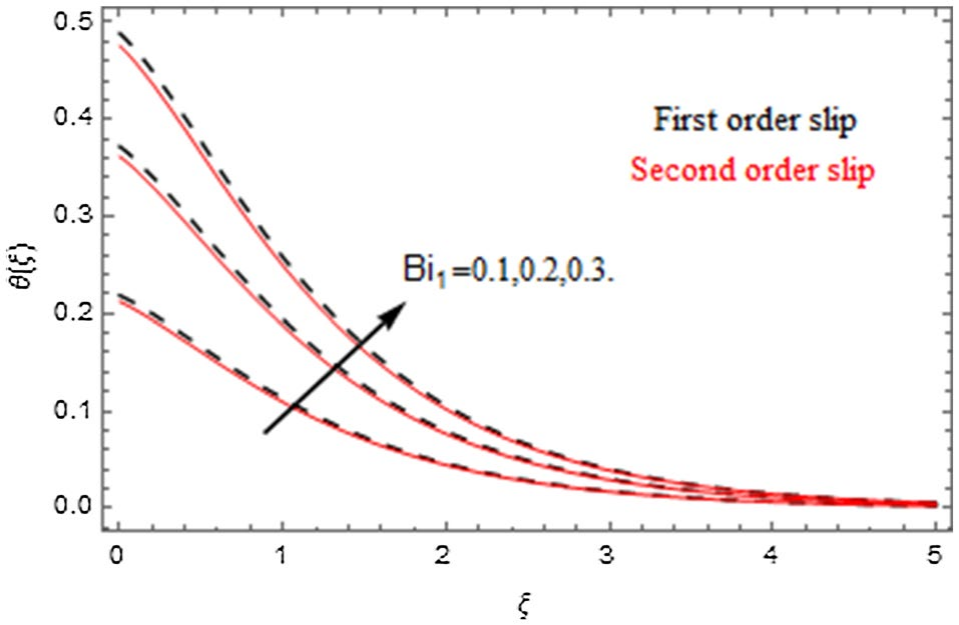
$$Bi_{1}$$ versus $$\theta \left( \xi \right)$$.

**Figure 16:**
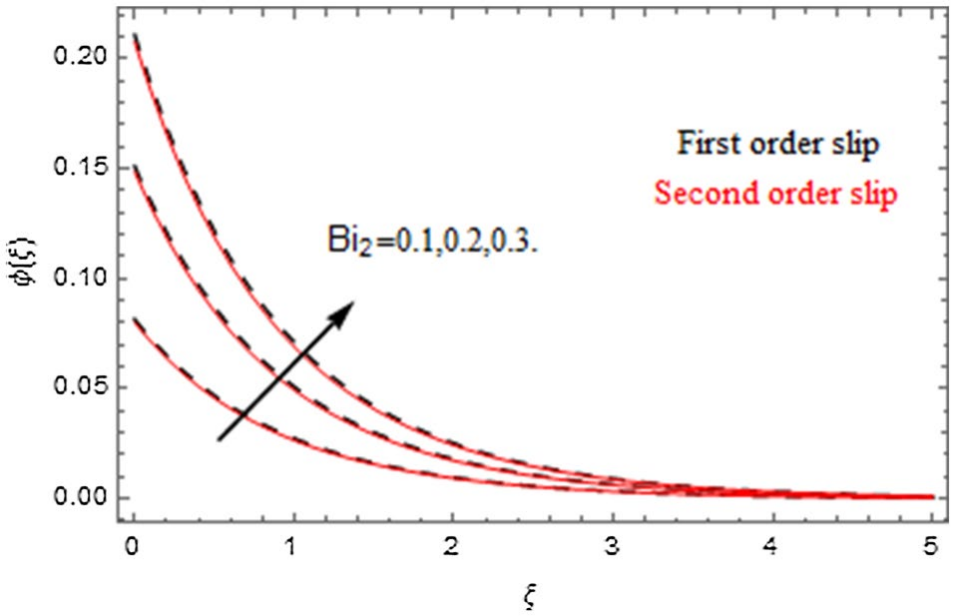
$$Bi_{2}$$ versus $$\phi \left( \xi \right)$$.

**Figure 17:**
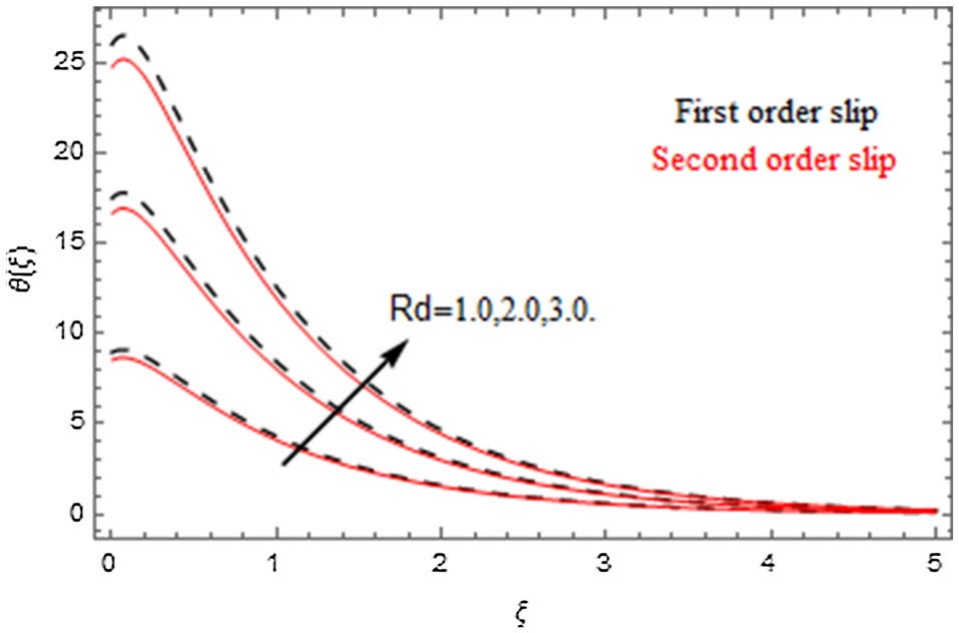
$$Rd$$ versus $$\theta \left( \xi \right)$$.

**Figure 18:**
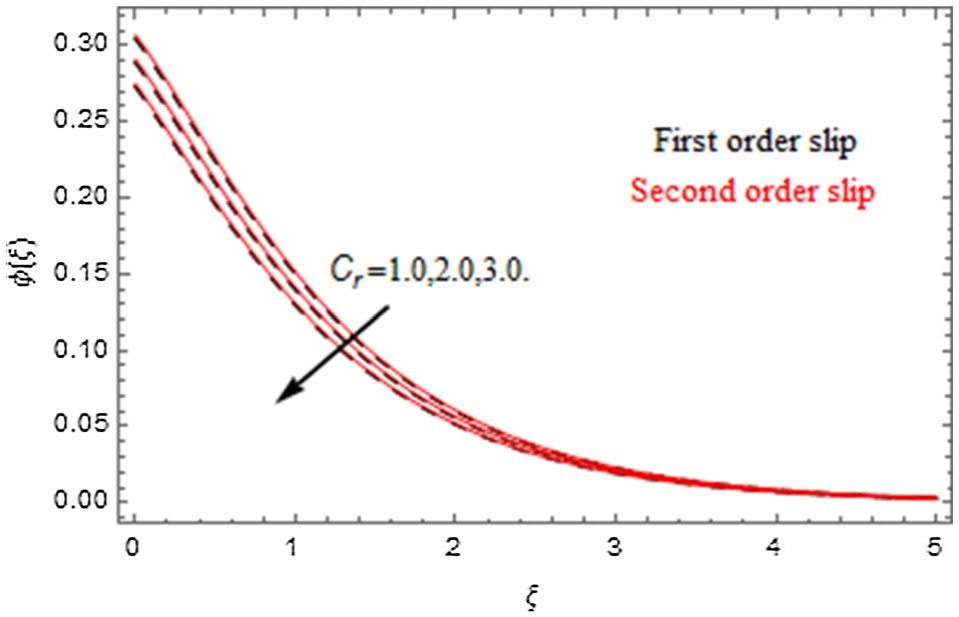
$$C_{r}$$ versus $$\phi \left( \xi \right)$$.

**Figure 19:**
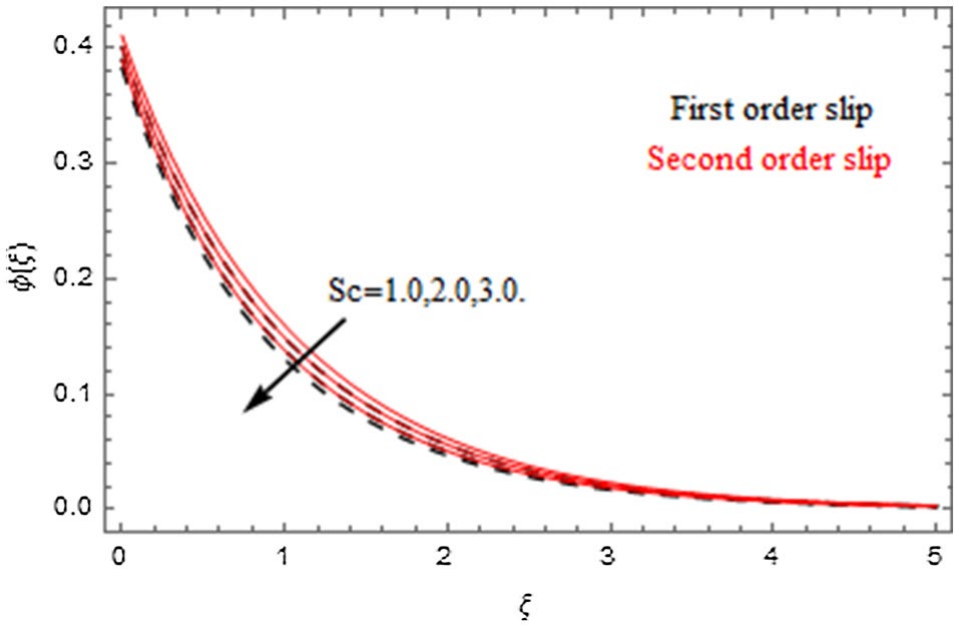
$$Sc$$ versus $$\phi \left( \xi \right)$$.

The impact of relevance parameters on velocity, micro-rotation, thermal, and concentration fields for first order slip $\gamma$ and second order slip $\delta$ parameters are offered in Figs. 5, 6, 7, 8, 9, 10, 11, 12, 13, 14, 15, 16, 17, 18 and 19. Figures 5 and 6 represent the effect of micropolar parameter on velocity and micro-rotation fields when $Rd = 0.3$, $\theta_{w} = 0.1$,$Bi_{1} = Bi_{2} = 0.5$, $\Pr = 7.0$, $\gamma = 0.5$, $\delta = 1.0$, $M = 1.0$, $C_{r} = 0.5$, $Sc = 1.0$, $\lambda = 0.2$, $A^{*} = B^{*} = 0.2$ and $M_{r} = 0.5$. The heightening in micropolar factor increases the velocity field however reduces the micro-rotation field. This findings indicate that the momentum exchange layer-by-layer is enhanced positively by the increase in viscosity induced by the mutual micro-rotation of the molecules, i.e. the significant estimates of $\alpha$; across the other side, the thermal diffusions are marginally weakened. Figures 7 and 8 present the influence of micro-rotation factor on velocity and micro-rotation fields when $Rd = 0.3$, $\alpha = 2.0$, $\theta_{w} = 0.1$, $Bi_{1} = Bi_{2} = 0.5$, $\Pr = 7.0$, $\gamma = 0.5$, $\delta = 1.0$, $M = 1.0$, $C_{r} = 0.5$, $Sc = 1.0$, $\lambda = 0.2$ and $A^{*} = B^{*} = 0.2$. The micro-rotation parameter has direct relation with velocity field, while has reverse relation with micro-rotation filed. The increasing micro-rotation parameter heightens the velocity field and has reducing impact on micro-rotation filed. In addition, it is also determined that there is no micro-rotation velocity when $Mr = 0$. Figure 9 indicates the relation between magnetic parameter and velocity field when $Rd = 0.3$, $\alpha = 2.0$, $\theta_{w} = 0.1$, $Bi_{1} = Bi_{2} = 0.5$, $\Pr = 7.0$, $C_{r} = 0.5$, $Sc = 1.0$, $\gamma = 0.5$, $\delta = 1.0$, $\lambda = 0.2$, $A^{*} = B^{*} = 0.2$ and $M_{r} = 0.5$. It contests the physical understanding of the contraction of the magnetic force to the electrically conductive fluid, yet this result in the increase of a drag force which resulted in the slowing down force on the velocity. Figure 10 designates the variation in velocity field against $\gamma$ and $\delta$ factors when $Rd = 0.3$, $\alpha = 2.0$, $C_{r} = 0.5$, $Sc = 1.0$, $\theta_{w} = 0.1$, $Bi_{1} = Bi_{2} = 0.5$, $\Pr = 7.0$, $M = 1.0$, $\lambda = 0.2$, $A^{*} = B^{*} = 0.2$ and $M_{r} = 0.5$. $\gamma$ and velocity field has inverse relation to each other. That is, the heightening in $\gamma$ the reduction in velocity field is detected. $\delta$ and velocity field has direct relation to each other. That is, the heightening in $\delta$ the rise in velocity field is detected. Figure 11 indicates the similar impact of $\gamma$ and $\delta$ against micro-rotation field when $Rd = 0.3$, $\alpha = 2.0$, $\theta_{w} = 0.1$, $Bi_{1} = Bi_{2} = 0.5$, $\Pr = 7.0$, $M = 1.0$, $\lambda = 0.2$, $C_{r} = 0.5$, $Sc = 1.0$, $A^{*} = B^{*} = 0.2$ and $M_{r} = 0.5$. Figures 12 and 13 display the impact of $A^{*}$ and $B^{*}$ on thermal field when $Rd = 0.3$, $\alpha = 2.0$, $\theta_{w} = 0.1$, $C_{r} = 0.5$, $Sc = 1.0$, $Bi_{1} = Bi_{2} = 0.5$, $\Pr = 7.0$, $\gamma = 0.5$, $\delta = 1.0$, $M = 1.0$, $\lambda = 0.2$ and $M_{r} = 0.5$. It is detected that the rising values $A^{*}$ and $B^{*}$ heighten the thermal field of the nanofluid flow. Actually, $A^{*}$ and $B^{*}$ act like a heat originator. The heightening estimations of $A^{*}$ and $B^{*}$ escalate the temperature of the fluid flow. Therefore, the increasing values of $A^{*}$ and $B^{*}$ intensifies the thermal field. Figure 14 depicts the impact of $\theta_{w}$ on thermal field when $Rd = 0.3$, $\alpha = 2.0$, $Bi_{1} = Bi_{2} = 0.5$, $\Pr = 7.0$, $C_{r} = 0.5$, $Sc = 1.0$, $\gamma = 0.5$, $\delta = 1.0$, $M = 1.0$, $\lambda = 0.2$, $A^{*} = B^{*} = 0.2$ and $M_{r} = 0.5$. With nonlinear radiation, the value of the temperature ratio parameter must be greater than 1. Also, a boost throughout the temperature ratio parameter allows the temperature to ruse through the layer. It is important to mention that as the temperature ratio tends to 1, the linear and non-linear Rosseland estimations have the same impact on the fluid flow temperature. The increasing influence of $\theta_{w}$ on temperature field is detected here. Figure 15 displays the impact of Biot number $Bi_{1}$ on thermal field when $Rd = 0.3$, $\alpha = 2.0$, $\theta_{w} = 0.1$, $Bi_{2} = 0.5$, $A^{*} = B^{*} = 0.2$, $C_{r} = 0.5$, $Sc = 1.0$, $\Pr = 7.0$, $\gamma = 0.5$, $\delta = 1.0$, $M = 1.0$, $\lambda = 0.2$ and $M_{r} = 0.5$. Biot number takes place in the special counsel investigation due to the implication of the convective boundary condition and tends to mean the proportion of both the diffuse opposition throughout the sheet to the convective opposition at the layer of the piece of paper. The lower estimations of the Biot number, therefore, provide an elevated convective opposition at the layer, and one that tends to lead to a medium heat transfer rate from layer to that of the liquid. So, the higher estimations of Biot number $Bi_{1}$ raise the temperature field of the fluid flow. A similar impact of Biot number $Bi_{2}$ against the concentration field when $Rd = 0.3$, $\alpha = 2.0$, $C_{r} = 0.5$, $Sc = 1.0$, $\theta_{w} = 0.1$, $Bi_{1} = 0.5$, $A^{*} = B^{*} = 0.2$, $\Pr = 7.0$, $\gamma = 0.5$, $\delta = 1.0$, $M = 1.0$, $\lambda = 0.2$ and $M_{r} = 0.5$ is shown in Fig. 16. Figure 17 signifies the non-linear thermal radiation $Rd$ impact on temperature field when $Rd = 0.3$, $\alpha = 2.0$, $\theta_{w} = 0.1$, $Bi_{1} = Bi_{2} = 0.5$, $A^{*} = B^{*} = 0.2$, $\Pr = 7.0$, $C_{r} = 0.5$, $Sc = 1.0$, $\gamma = 0.5$, $\delta = 1.0$, $M = 1.0$, $\lambda = 0.2$ and $M_{r} = 0.5$. The rise in $Rd$ escalates the temperature field. It is well known that the function of radiation and thermal expansion is indeed a phenomenon that creates heat through fluid particles in such a manner that certain extra heat is created all through the flow. Figure 18 denotes the influence of $C_{r}$ on concentration field when $Rd = 0.3$, $\alpha = 2.0$, $\theta_{w} = 0.1$, $Bi_{1} = Bi_{2} = 0.5$, $Sc = 1.0$, $A^{*} = B^{*} = 0.2$, $\Pr = 7.0$, $\gamma = 0.5$, $\delta = 1.0$, $M = 1.0$, $\lambda = 0.2$ and $M_{r} = 0.5$. Higher values of the chemical reaction parameter lead to a greater rate of pernicious chemical change that breaks down or halts the liquid species more efficiently and effectively. Thus a decreasing influence in concentration profile is depicted. Figure 19 signifies the association of $Sc$ with concentration field when $Rd = 0.3$, $\alpha = 2.0$, $\theta_{w} = 0.1$, $Bi_{1} = Bi_{2} = 0.5$, $A^{*} = B^{*} = 0.2$, $\Pr = 7.0$, $\gamma = 0.5$, $\delta = 1.0$, $C_{r} = 0.5$, $M = 1.0$, $\lambda = 0.2$ and $M_{r} = 0.5$. The Schmidt number is inversely related with concentration field. An increase in $Sc$ deescalates the concentration field. It is evidently understood that concentration, in addition to its related boundary layer thickness, are diminishing functions of $Sc$. The improvement in $Sc$, thus leads to a lower coefficient of diffusion. Such a lower coefficient of diffusion results a significant decrease throughout the concentration field.

**Figure 20:**
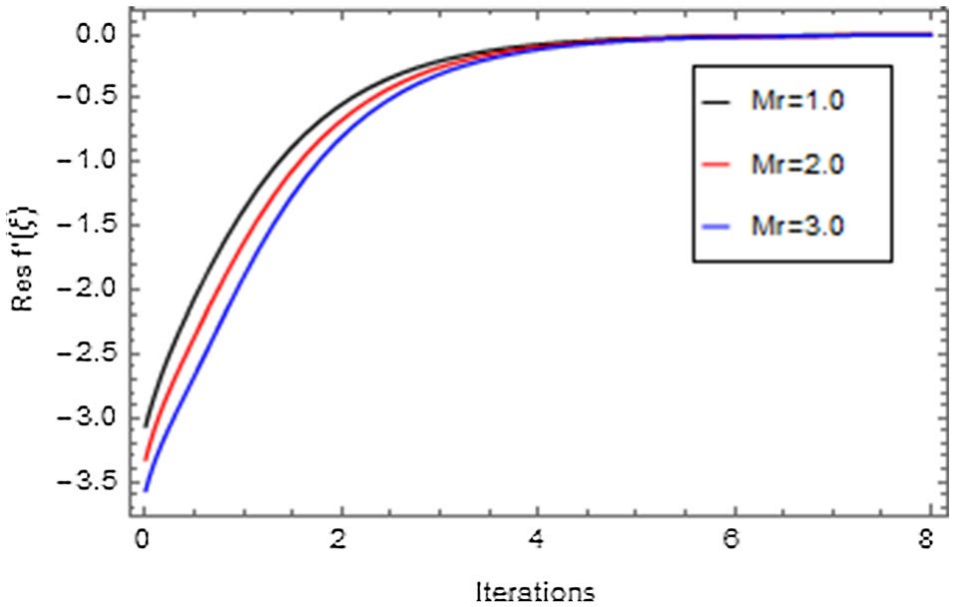
Residual error for $Mr$ on $f^{\prime}\left( \xi \right)$.

**Figure 21:**
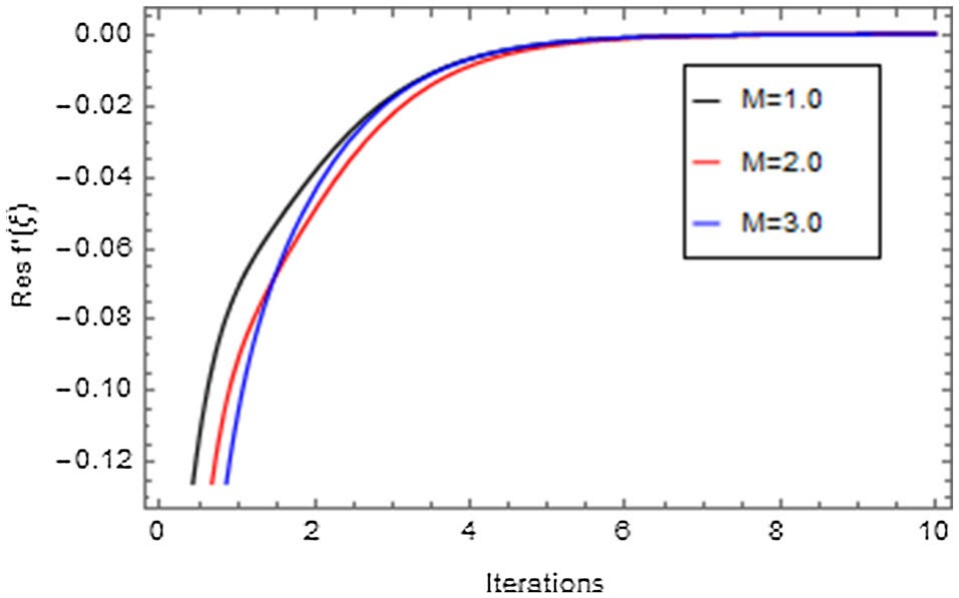
Residual error for $M$ on $f^{\prime}\left( \xi \right)$.

**Figure 22:**
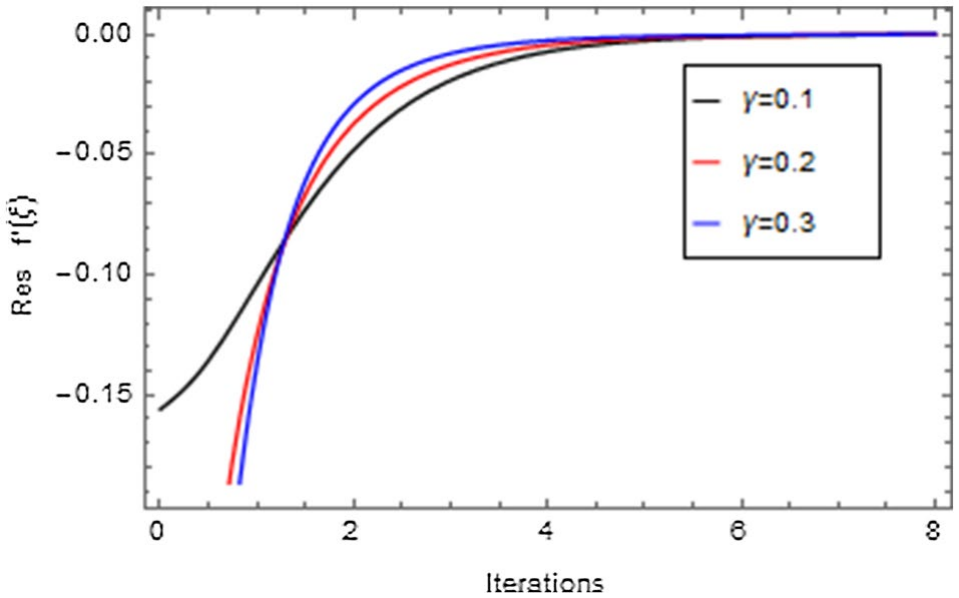
Residual error for $\gamma$ on $f^{\prime}\left( \xi \right)$.

**Figure 23:**
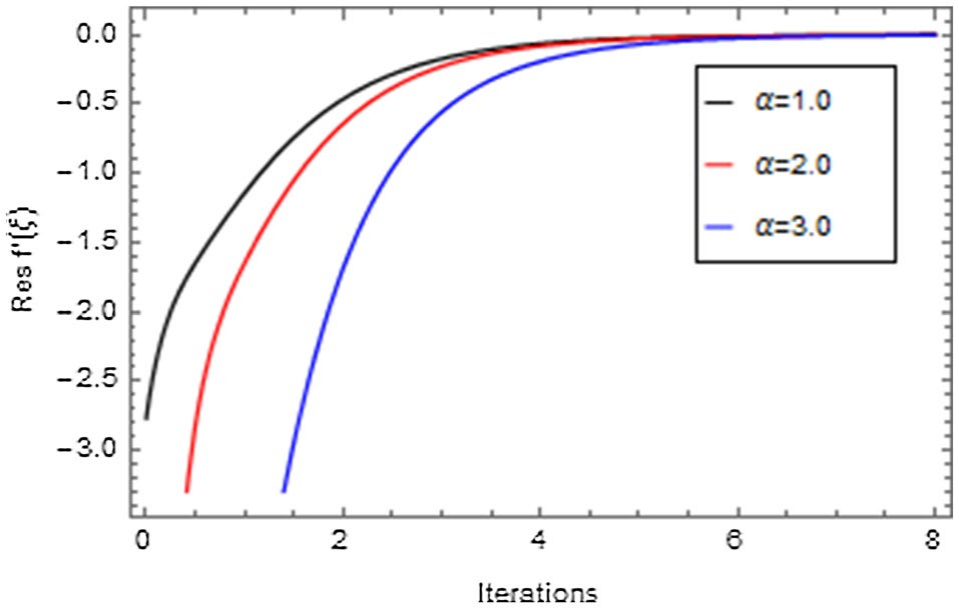
Residual error for $\alpha$ on $f^{\prime}\left( \xi \right)$.

**Figure 24:**
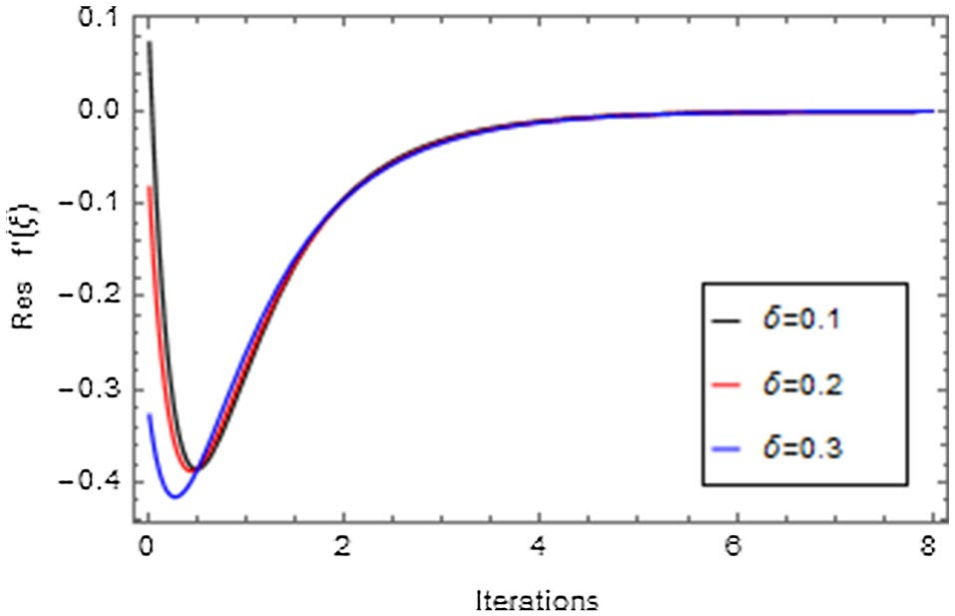
Residual error for $\delta$ on $f^{\prime}\left( \xi \right)$.

**Figure 25:**
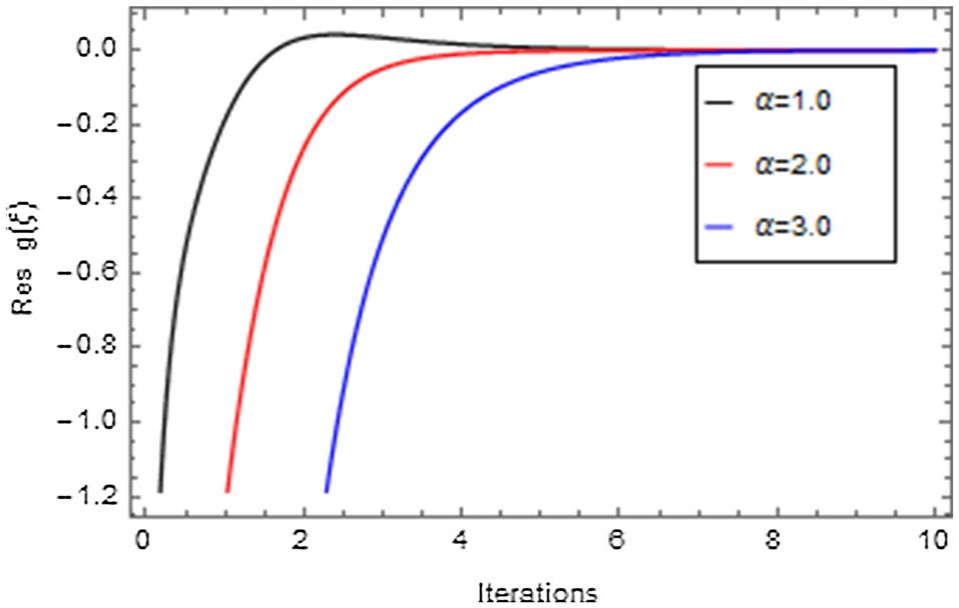
Residual error for $\alpha$ on $g\left( \xi \right)$.

**Figure 26:**
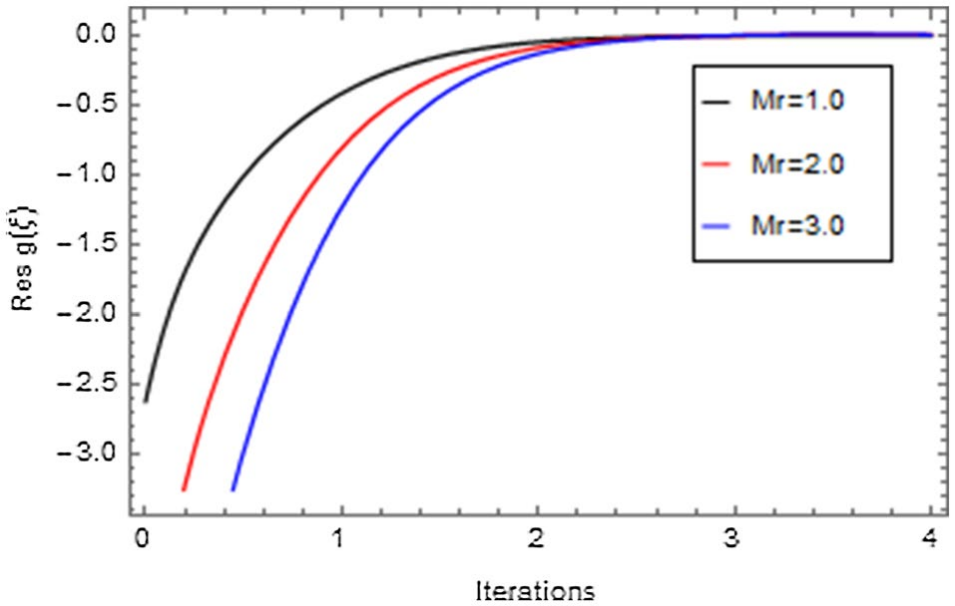
Residual error for $Mr$ on $g\left( \xi \right)$.

**Figure 27:**
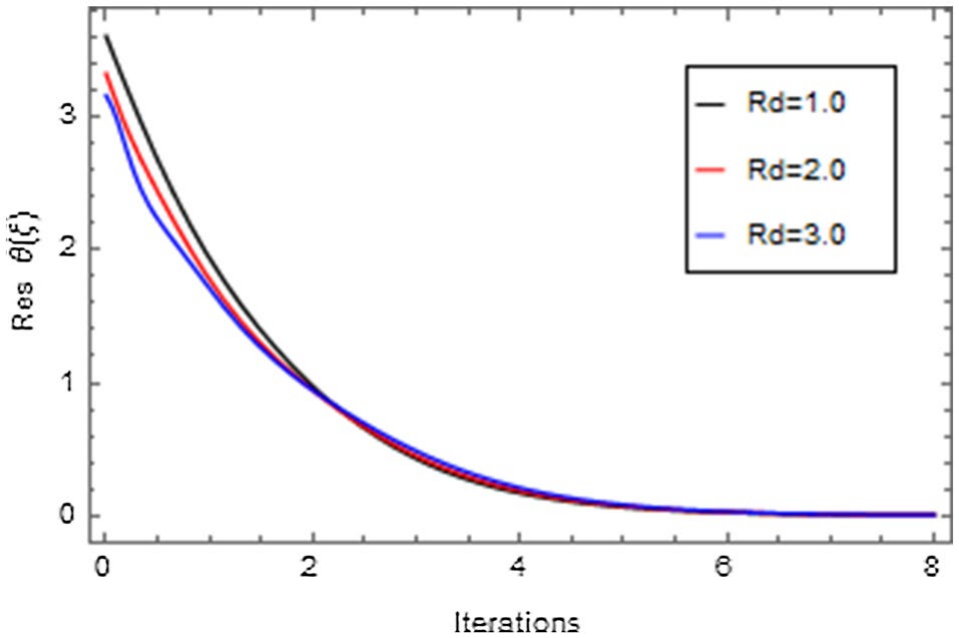
Residual error for $Rd$ on $\theta \left( \xi \right)$.

**Figure 28:**
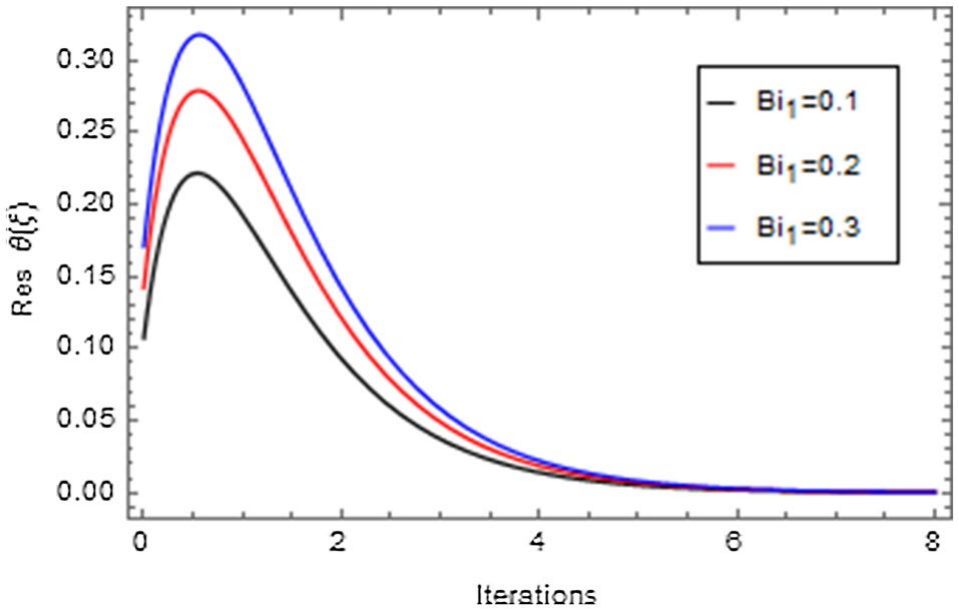
Residual error for $Bi_{1}$ on $\theta \left( \xi \right)$.

**Figure 29:**
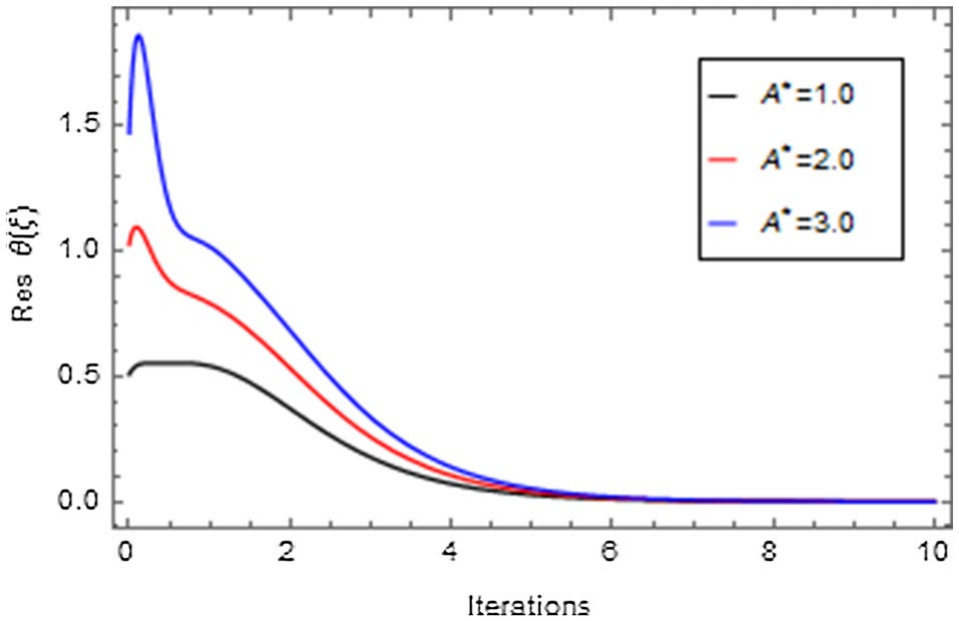
Residual error for $A^{*}$ on $\theta \left( \xi \right)$.

**Figure 30:**
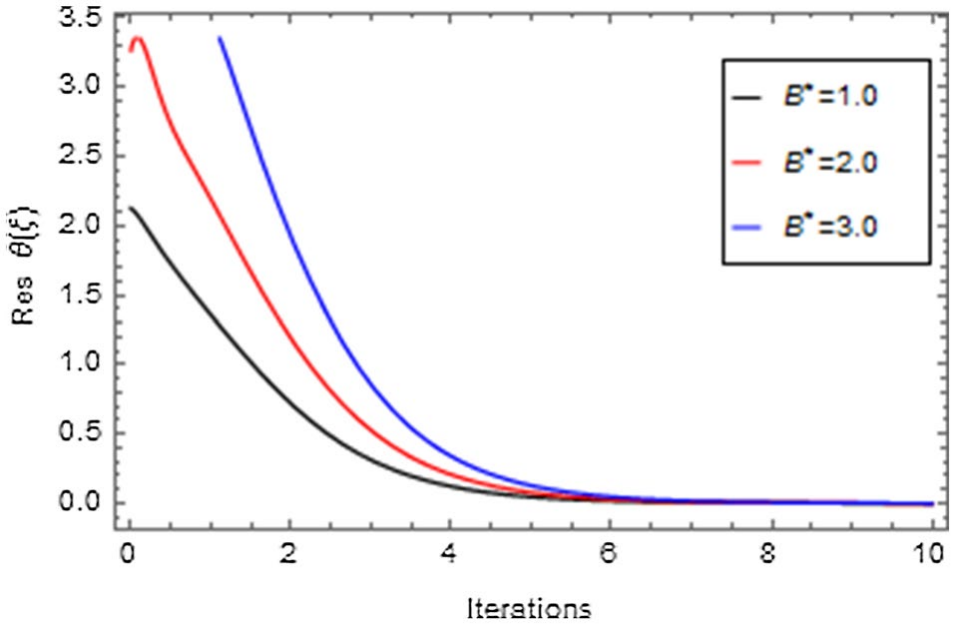
Residual error for $B^{*}$ on $\theta \left( \xi \right)$.

**Figure 31:**
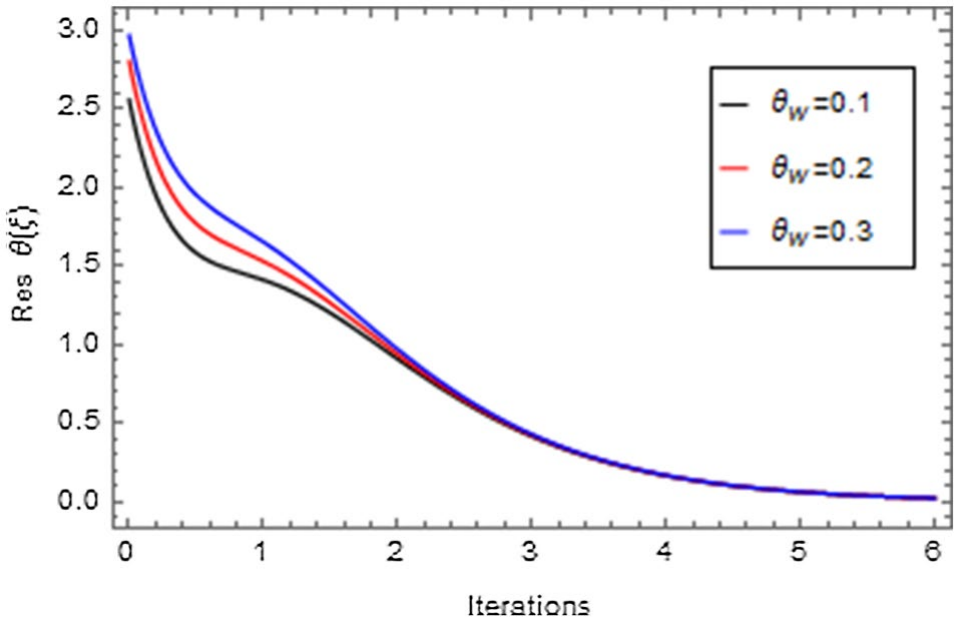
Residual error for $\theta_{w}$ on $\theta \left( \xi \right)$.

**Figure 32:**
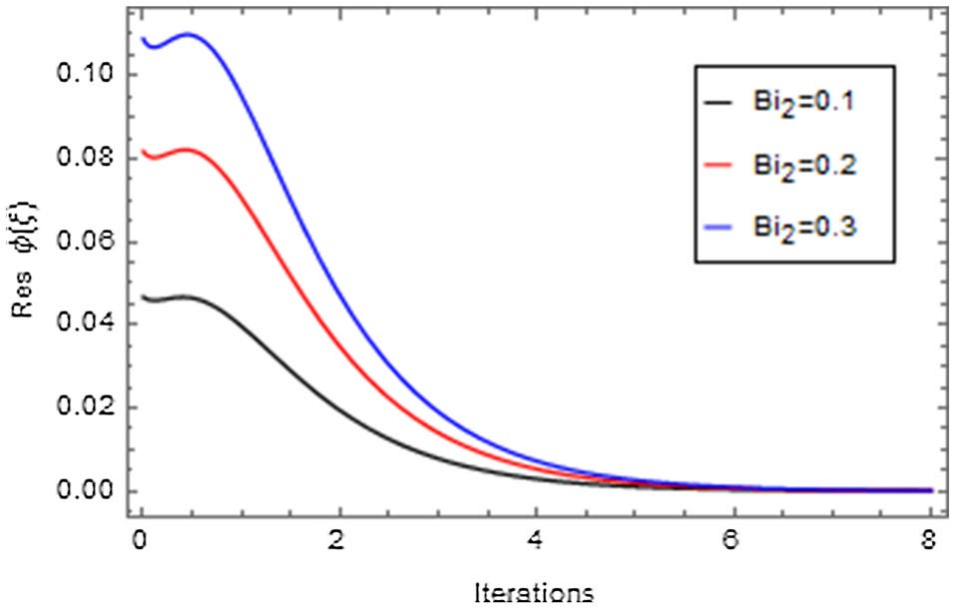
Residual error for $Bi_{2}$ on $\phi \left( \xi \right)$.

**Figure 33:**
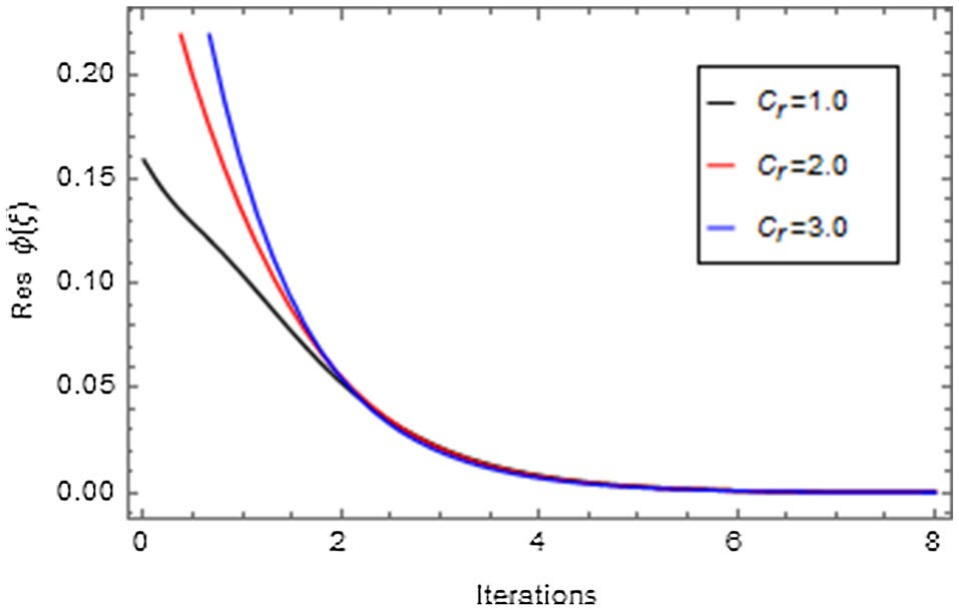
Residual error for $C_{r}$ on $\phi \left( \xi \right)$.

**Figure 34:**
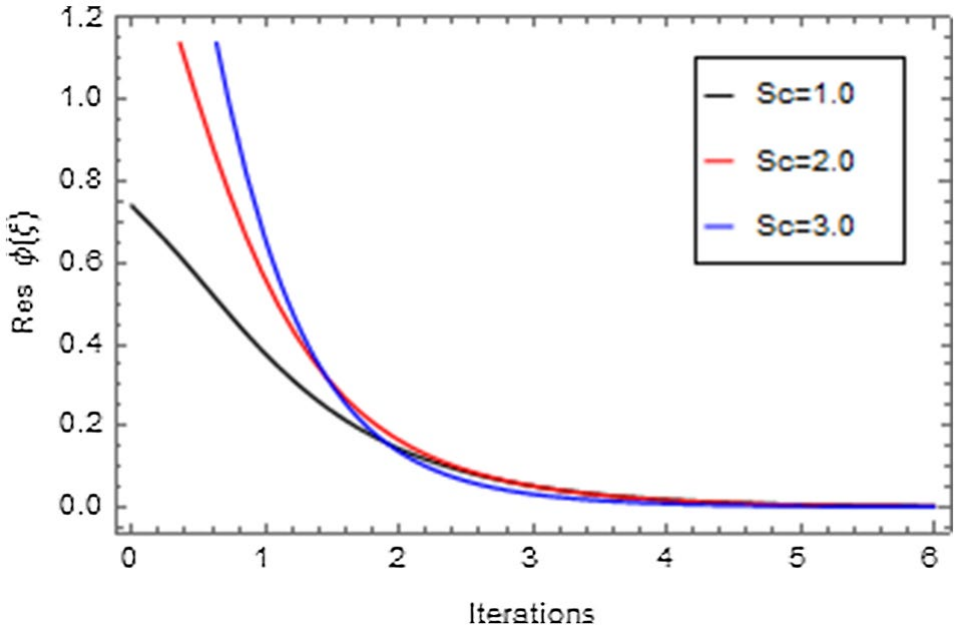
Residual error for $Sc$ on $\phi \left( \xi \right)$.

Figures 20, 21, 22, 23, 24, 25, 26, 27, 28, 29, 30, 31, 32, 33 and 34 are displayed to examine the fitting deviation or residual error for velocity, micro-rotation, temperature, and concentration profiles via influential parameters for the persistence of defining the correctness and determining the convergence of solution method. The residual errors for velocity profile $f^{\prime}\left( \xi \right)$ via $Mr$, $M$, $\gamma$, $\alpha$ and $\delta$ are displayed in Figs. 20, 21, 22, 23, and 24. The residual error for $Mr$ converges quickly after 0.0 < iteration < 5.0 which is shown in Fig. 20. This effect shows the correctness and convergence of the activated technique (HAM). Figure 21 displays the residual error for $M$ on $f^{\prime}\left( \xi \right)$. The residual error for $M$ converges speedily after 0.0 < iteration < 6.0. Figures 22, 23, and 24 indicate the residual errors for $\gamma$, $\alpha$ and $\delta$ on $f^{\prime}\left( \xi \right)$, respectively. The residual errors for $\gamma$, $\alpha$ and $\delta$ rapidly converges after 0.0 < iteration < 5.5, 0.0 < iteration < 5.5, 0.0 < iteration < 6.0 and 0.0 < iteration < 4.0, respectively. The residual errors for micro-rotation profile $g\left( \xi \right)$ via $\alpha$ and $Mr$ are displayed in Figs. 25 and 26 respectively. The residual error for $\alpha$ converges quickly after 0.0 < iteration < 7.5. Also the residual error for $Mr$ converges quickly for 0.0 < iteration < 2.8. The residual errors for thermal profile $\theta \left( \xi \right)$ via $Rd$, $Bi_{1}$, $A^{*}$,$B^{*}$ and $\theta_{w}$ are displayed in Figs. 27, 28, 29, 30 and 31. The residual error for $Rd$ converges quickly after 0.0 < iteration < 6.0 which is shown in Fig. 27. The similar effect of $Bi_{1}$ is observed in Fig. 28. The residual errors for $\theta \left( \xi \right)$ via $A^{*}$ and $B^{*}$ are displayed in Figs. 29 and 30 respectively. The residual errors converge quickly after 0.0 < iteration < 5.0. The residual error for $\theta_{w}$ converges quickly for 0.0 < iteration < 4.5 which is displayed in Fig. 31. The residual errors for concentration profile $\phi \left( \xi \right)$ via $Bi_{2}$, $C_{r}$ and $Sc$ are displayed in Figs. 32, 33, and 34. The residual errors of $Bi_{2}$, $C_{r}$ and $Sc$ are quickly converge after 0.0 < iteration < 7.0, 0.0 < iteration < 6.0 and 0.0 < iteration < 5.0, respectively.

**Figure 35:**
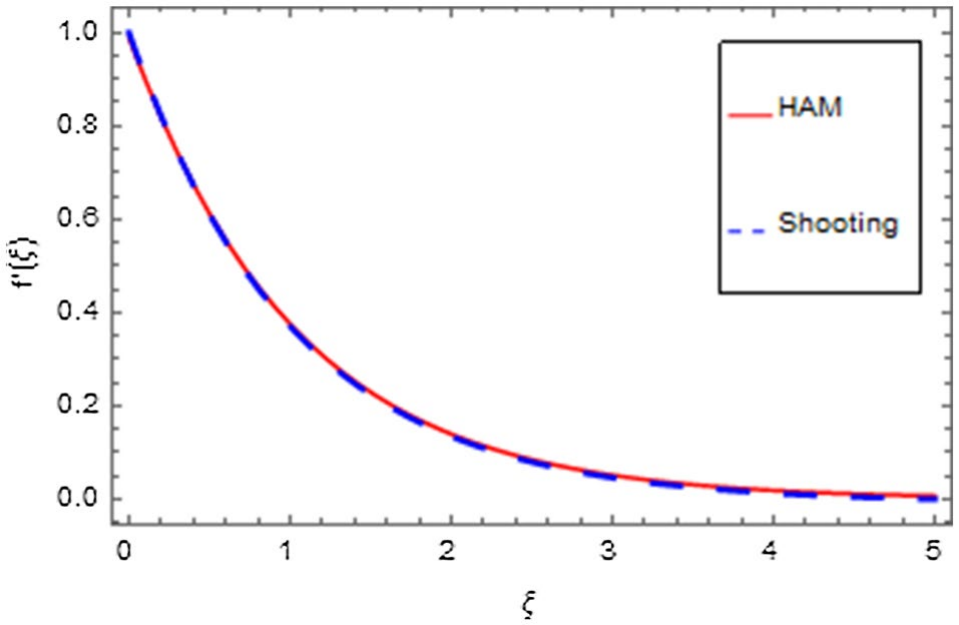
HAM versus shooting for $f^{\prime}\left( \xi \right)$.

**Figure 36:**
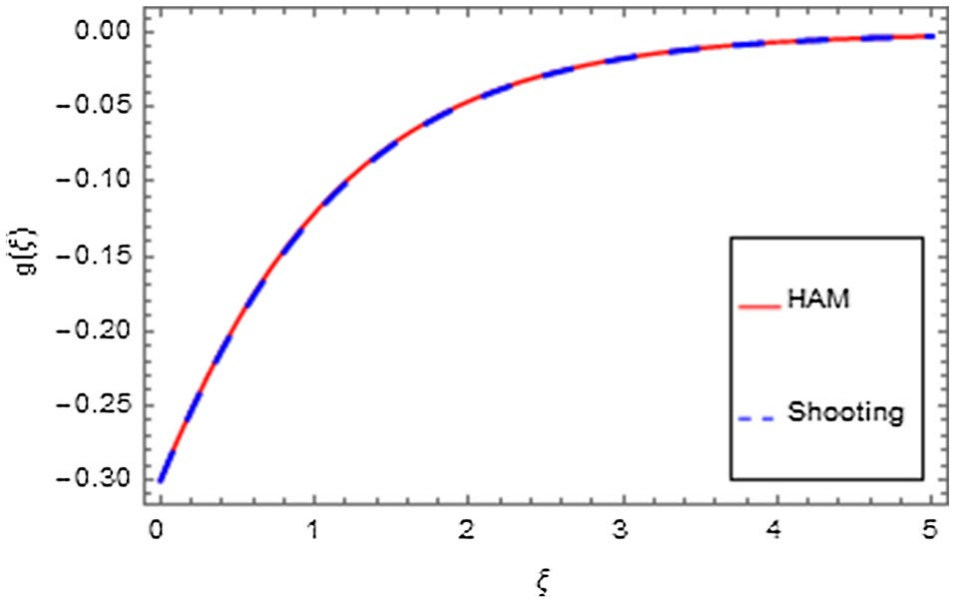
HAM versus shooting for $g\left( \xi \right)$.

**Figure 37:**
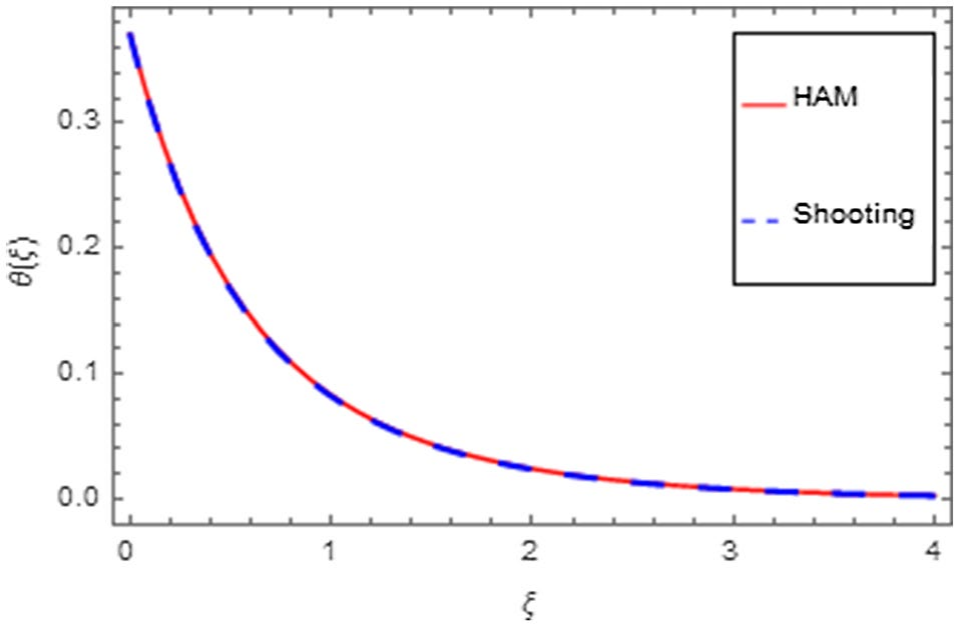
HAM versus shooting for $\theta \left( \xi \right)$.

**Figure 38:**
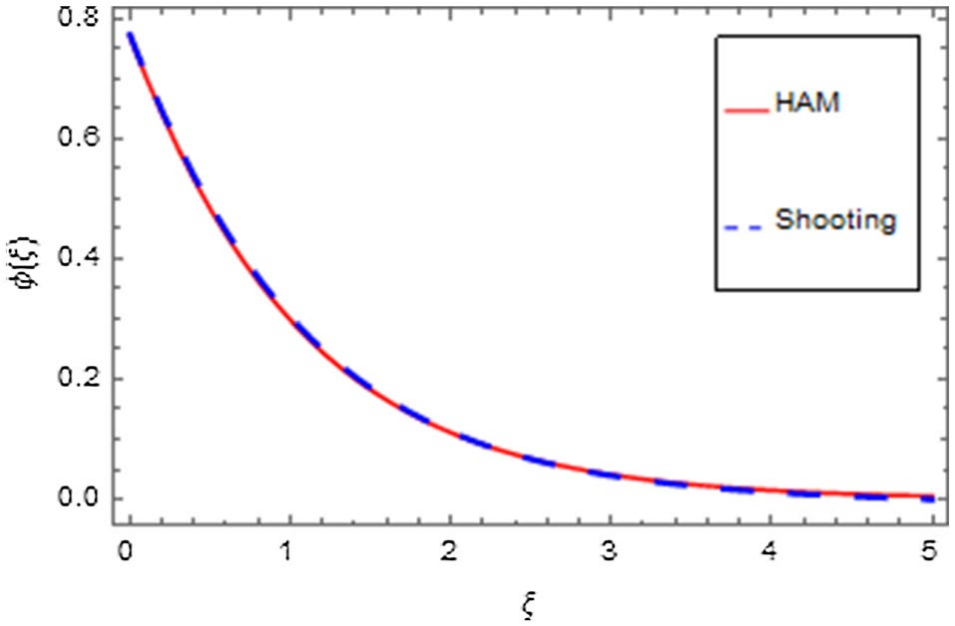
HAM versus shooting for $\phi \left( \xi \right)$.

An analytical solution is the actual solution. An analytical approach with varying properties can be used to analyze the behavior of systems. Unfortunately, there are many functional methods that relate to an analytical solution, and analytical methods are mostly of minimal use. That's why we have used a numerical method to produce response that seems to be closer to realistic outcomes. In the physical world, there are virtually no problems that can be perfectly solved, making the problem more complicated than other problems that can be exactly solved. There are three or four of them in existence that have already been resolved, but nevertheless still numerical approaches do not always provide an efficient solution. Numerical methods can be applied to any finalized physical geometry that is sometimes hard to address analytically. Here we have applied both analytical and numerical approaches to solve the modeled system of equations. Both techniques have quite close agreement as shown in Figs. 35, 36, 37 and 38 and Tables 1, 2, 3 and 4.

**Table 1:** HAM versus shooting for $f^{\prime}\left( \xi \right)$.

$\xi$	HAM	Shooting
0.0	− 2.42861 × 10^–17^	0.000000
0.5	0.393406	0.394616
1.0	0.635715	0.634836
1.5	0.784043	0.780444
2.0	0.879508	0.868196
2.5	0.929562	0.920581
3.0	0.963022	0.951360
3.5	0.983341	0.968874
4.0	0.997526	0.978229
4.5	1.003160	0.982535
5.0	1.007700	0.983686

**Table 2:** HAM versus shooting for $g\left( \xi \right)$.

$\xi$	HAM	Shooting
0.0	− 0.300000	− 0.300000
0.5	− 0.194235	− 0.192929
1.0	− 0.122029	− 0.120811
1.5	− 0.075511	− 0.074624
2.0	− 0.046340	− 0.045751
2.5	− 0.028303	− 0.027027
3.0	− 0.017238	− 0.017003
3.5	− 0.010482	− 0.010337
4.0	− 0.006367	− 0.006278
4.5	− 0.003865	− 0.003811
5.0	− 0.002345	− 0.002312

**Table 3:** HAM versus shooting for $\theta \left( \xi \right)$.

$\xi$	HAM	Shooting
0.0	0.188612	0.221368
0.5	− 0.016859	0.015829
1.0	− 0.049618	− 0.026464
1.5	− 0.042942	− 0.027918
2.0	− 0.030446	− 0.021020
2.5	− 0.020068	− 0.014194
3.0	− 0.012698	− 0.009128
3.5	− 0.007903	− 0.005725
4.0	− 0.004867	− 0.003541
4.5	− 0.002978	− 0.002172
5.0	− 0.001816	− 0.001327

**Table 4:** HAM versus shooting for $\phi \left( \xi \right)$.

$\xi$	HAM	Shooting
0.0	0.768626	0.772196
0.5	0.482797	0.489327
1.0	0.297773	0.303516
1.5	0.182298	0.185537
2.0	0.111148	0.112200
2.5	0.067617	0.067150
3.0	0.041084	0.039587
3.5	0.024945	0.022635
4.0	0.015139	0.012001
4.5	0.009186	0.005016
5.0	0.005572	− 1.4124 × 10^–26^

The assessment of skin factor, heat, and mass transfer rates are calculated in Tables 5, 6, 7 and 8. Table 5 represents the influence of corresponding parameters on skin factor $C_{f}$. The higher estimations of micropolar parameter reduce $C_{f}$ whereas the larger values of magnetic parameter escalate $C_{f}$. Also, the present study in compared with Kumar et al.^43^ and has agreed with the past analysis. Table 6 expresses the assessments of $C_{f}$ and $C_{s}$ against $\delta = 0$ and $\delta = 1$ for different values of the corresponding factors. $C_{f}$ escalates for higher estimations of micropolar and micro-rotation parameters, while this behavior is opposite against magnetic parameter. $C_{s}$ diminishes for higher values of micropolar, micro-rotation and magnetic field parameters. Table 7 illustrates the assessments of heat transfer rate $Nu$ against $\delta = 0$ and $\delta = 1$ for unalike estimations of the corresponding factors. The higher estimations of heat source and sink, temperature ratio parameter and non-linear thermal energy parameter deescalates $Nu$, while the Biot number $Bi_{1}$ has inverse influence via $Nu$. Table 8 signifies assessment of $Sh$ against $\delta = 0$ and $\delta = 1$ for different values of the corresponding factors. The higher estimations of Biot number $Bi_{2}$ and chemical reaction parameter hikes the $Sh$ while the Schmidt number reduces $Sh$.

**Table 5:** Judgment of the skin friction $C_{f}$ of the present analysis with previous investigation against different estimations of $\alpha$ and $M$.

$\alpha$	$M$	Kumar et al.^43^	Present analysis
1.0		0.31709	0.317097
2.0		0.30676	0.306764
3.0		0.29713	0.297135
4.0		0.28841	0.288410
	0.1	0.32196	0.321963
	0.2	0.32623	0.326239
	0.3	0.32933	0.329332

**Table 6:** Assessments of $C_{f}$ and $C_{s}$ against different values of the corresponding factors.

	$C_{f}$		$C_{s}$	
	$\delta = 0$	$\delta = 1$	$\delta = 0$	$\delta = 1$
$\alpha = 1.0$	− 0.600453	− 0.820725	− 0.306835	− 0.429662
$\alpha = 2.0$	− 0.566971	− 0.725193	− 0.275236	− 0.358507
$\alpha = 3.0$	− 0.540782	− 0.664839	− 0.246085	− 0.307275
$M_{r} = 1.0$	− 0.270378	− 0.276755	0.595872	0.610186
$M_{r} = 2.0$	− 0.239194	− 0.230289	0.632875	0.682285
$M_{r} = 3.0$	− 0.215495	− 0.197424	0.732696	0.767432
$M = 2.0$	− 0.315575	− 0.346335	0.058832	0.064125
$M = 3.0$	− 0.342973	− 0.392325	0.051585	0.058475
$M = 5.0$	− 0.359486	− 0.426532	0.045825	0.053846

**Table 7:** Assessment of $Nu$ against different values of the corresponding factors.

	$$Nu$$	
	$\delta = 0$	$\delta = 1$
$A^{*} = 1.0$	0.855429	0.836741
$A^{*} = 2.0$	0.739836	0.742832
$A^{*} = 3.0$	0.420749	0.423428
$B^{*} = 1.0$	0.457476	0.397397
$B^{*} = 2.0$	0.123974	0.125165
$B^{*} = 3.0$	0.096440	0.096542
$\theta_{w} = 1.0$	0.336174	0.339975
$\theta_{w} = 2.0$	0.328617	0.329640
$\theta_{w} = 3.0$	0.326418	0.328393
$Bi_{1} = 0.1$	0.107880	0.158824
$Bi_{1} = 0.2$	0.184497	0.185759
$Bi_{1} = 0.3$	0.252874	0.254822
$Rd = 1.0$	0.348153	0.350865
$Rd = 2.0$	0.345652	0.347967
$Rd = 3.0$	0.342979	0.345142

**Table 8:** Assessment of $Sh$ against different values of the corresponding factors.

	$$Sh$$	
	$\delta = 0$	$\delta = 1$
$Bi_{2} = 0.1$	0.481612	0.481265
$Bi_{2} = 0.2$	0.634121	0.634742
$Bi_{2} = 0.3$	0.708909	0.709680
$C_{r} = 1.0$	0.715317	0.716092
$C_{r} = 2.0$	0.531242	0.732006
$C_{r} = 3.0$	0.747005	0.747758
$Sc = 1.0$	0.857845	0.860115
$Sc = 2.0$	1.002340	1.006230
$Sc = 3.0$	1.131470	1.136380

## Conclusion

The electrically accompanying magnetohydrodynamic micropolar nanofluid flow over an extending sheet with secondary slips conditions and chemical reaction is considered here. The nanofluid flow is considered in two dimensional coordinates system. The proposed model is treated analytically and numerically. In order to improve the validity of the solutions and the method convergence, a descriptive demonstration of residual errors for various factors is presented. The main results are set out below.

- The velocity field heightens with the rise in micropolar factor, micro-rotation factor and primary velocity factor whereas reduces with escalation in magnetic factor, and secondary velocity slip parameter.
- The micro-rotation field rises with the escalation in primary order velocity slip factor while reduces with micro-rotation parameter, secondary order velocity slip parameter, and micropolar factor.
- The thermal field heightens with escalating non-uniform heat sink/source, Biot number, temperature ratio factor, and thermal radiation factor.
- The concentration field escalates with the increasing Biot number, while reduces with heightening chemical reaction and Schmidt number.
- Analytical and numerical approaches have quite close agreement.

## List of symbols

$$c,\,\,d$$: Constants
$$\kappa$$: Vertex viscosity
$B_{0}$: Magnetic field strength
$$\mu$$: Dynamic viscosity
$$\rho$$: Density
$$\sigma$$: Electrical conductivity
$$\rho c_{p}$$: Heat capacitance
$$k$$: Thermal conductivity
$Mr$: Micro-rotation parameter
$j = {\upsilon \mathord{\left/ {\vphantom {\upsilon c}} \right. \kern-\nulldelimiterspace} c}$: Micro-inertia density
$$\alpha = {\kappa \mathord{\left/ {\vphantom {\kappa \mu }} \right. \kern-\nulldelimiterspace} \mu }$$: Material parameter
$h_{f}$: Heat transfer coefficient
$h_{s}$: Mass transfer coefficient
$$K_{n}$$: Knudsen number
$\lambda$: Molecular free path
$$M$$: Magnetic factor
$$M_{r}$$: Microrotation parameter
$C_{r}$: Chemical reaction parameter
$\gamma$, $\delta$: Velocity slip factors
$$C_{f}$$: Skin friction
$${\text{Re}}_{x}$$: Reynolds number
$$\hbar_{f}$$,$$\hbar_{g}$$,$$\hbar_{\theta }$$,$$\hbar_{\phi }$$: Auxiliary factors
$$Sh$$: Sherwood number
$$x,\,\,y$$: Coordinates
$$u,\,\,v$$: Velocity components
$$D$$: Diffusion coefficient
$$K_{1}$$: Reaction rate
$N$: Micro-rotation velocity
$$T$$: Temperature
$T_{s}$: Temperature at the surface
$$T_{\infty }$$: Ambient temperature
$$C$$: Concentration
$$C_{s}$$: Surface concentration
$$C_{\infty }$$: Ambient concentration
$A^{*}$, $B^{*}$: Non-uniform heat source and sink parameters
$$P$$, $$Q$$: Constants
$$a$$: Momentum coefficient
$$\alpha$$: Micropolar factor
$$\Pr$$: Prandtl number
$Rd$: Non-linear thermal energy factor,
$$Bi_{1}$$, $$Bi_{2}$$: Biot numbers
$$\lambda$$: Stretching factor
$$Nu$$: Nusselt number
$a_{i} (i = 1 - 9)$: Constants in general solution
Res: Residual error
$$C_{s}$$: Couple stress
